# Hormone replacement therapy and cancer survival: a longitudinal cohort study: protocol paper

**DOI:** 10.1136/bmjopen-2020-046701

**Published:** 2021-08-02

**Authors:** Tom Alan Ranger, Judith Burchardt, Ashley Kieran Clift, Winnie Xue Mei, Carol Coupland, Pui San Tan, Sharon Dixon, Christopher Robert Cardwell, Julia Hippisley-Cox

**Affiliations:** 1 Nuffield Department of Primary Care Health Sciences, University of Oxford, Oxford, UK; 2 Division of Primary Care, University of Nottingham, Nottingham, UK; 3 School of Medicine, Dentistry and Biomedical Sciences, Queen’s University Belfast, Belfast, UK

**Keywords:** epidemiology, oncology, sex steroids & HRT

## Abstract

**Introduction:**

Hormone replacement therapy (HRT) can help women experiencing menopausal symptoms, but usage has declined due to uncertainty around risks of cancer and some cardiovascular diseases (CVD). Moreover, improved cancer survival rates mean that more women who survive cancer go on to experience menopausal symptoms. Understanding these relationships is important so that women and their clinicians can make informed decisions around the risks and benefits of HRT. This study’s primary aim is to determine the association between HRT use after cancer diagnosis and the risk of cancer-specific mortality. The secondary aims are to investigate the risks of HRT on subsequent cancer, all-cause mortality and CVD.

**Methods and analysis:**

We will conduct a population-based longitudinal cohort study of 18–79 year-old women diagnosed with cancer between 1998 and 2020, using the QResearch database. The main exposure is HRT use, categorised based on compound, dose and route of administration, and modelled as a time-varying covariate. Analysis of HRT use precancer and postcancer diagnosis will be conducted separately. The primary outcome is cancer-specific mortality, which will be stratified by cancer site. Secondary outcomes include subsequent cancer diagnosis, CVD (including venous thrombo-embolism) and all-cause mortality. Adjustment will be made for key confounders such as age, body mass index, ethnicity, deprivation index, comorbidities, and cancer grade, stage and treatment. Statistical analysis will include descriptive statistics and Cox proportional hazards models to calculate HRs and 95% CIs.

**Ethics and dissemination:**

Ethical approval for this project was obtained from the QResearch Scientific Committee (Ref: OX24, project title ‘Use of hormone replacement therapy and survival from cancer’). This project has been, and will continue to be, supported by patient and public involvement panels. We intend to the submit the findings for peer-reviewed publication in an academic journal and disseminate them to the public through Cancer Research UK.

**Strengths and limitations of this study**

- This is an open cohort study comprising a nationally representative sample of English women.
- The cohort consists of General Practice (GP) clinic data linked to hospital records, the English national cancer registry and English national death registry.
- This study has access to detailed information on hormone replacement therapy prescriptions, allowing analysis with consideration of the specific compound, dose, route of administration and duration of exposure.
- This study is limited by high rates of missing data for cancer grade and stage, although completeness has improved in recent years, this will be accounted for using appropriate multiple imputation techniques.

## Background

### Prevalence of HRT use

Hormone replacement therapy (HRT) involves clinical provision of the female sex hormone oestrogen either on its own or in combination with another hormone, progestogen. It is an effective and important treatment for symptoms of the menopause, including vasomotor symptoms (hot flushes and night sweats), urogenital atrophy^1^ and postmenopausal osteoporosis.^2^ Yet between 2002 and 2005, HRT usage in the UK dropped from approximately 29% of postmenopausal women to approximately 11%,^3^ and this pattern was consistent across Europe.^4^ The drop in usage has been attributed to the widely reported findings of the Million Women’s Study^5^ and the early cessation of the Women’s Health Initiative trial,^3 4 6^ which concluded that an observed increase in risk of cardiovascular events and breast cancer in women taking combined oestrogen and progestogen HRT outweighed the benefits of reduced rates of colorectal cancer and osteoporotic fractures.^7^ Evidence from a recent Cochrane systematic review and meta-analysis though, suggests that HRT remains an important treatment option for women with severe menopausal symptoms who do not have other risk factors, such as oestrogen sensitive cancers.^8^ Given the demonstrated risks and benefits of HRT, there is a need to clarify which women can use it safely, to treat menopausal symptoms without unduly increasing their risk of adverse outcomes.

### HRT in women with cancer

The role of oestrogen in cancer is complex, with evidence that it accelerates cancer progression in some sites but slows it in others. For instance, preclinical studies have shown that oestrogen stimulates growth in bladder^9^ and gastric cancer cell lines^10^ but, in contrast, similar studies found that it inhibits cell growth and/or induces apoptosis in oesophageal adenocarcinoma^11^ and malignant melanoma cell lines.^12^ What’s more, in a mouse model oestrogen increased the number and volume of lung adenocarcinomas,^13^ and molecular pathological studies have shown oestrogen receptor expression is associated with metastasis and poorer survival in gastric cancer.^14^ Epidemiological studies also report contrasting findings depending the cancer of interest, with some showing markedly lower incidence and slightly better survival for women compared with men for many cancers,^15 16^ whereas another study demonstrated survival after gastric cancer is worse in young women (<45 years old) than in young men, but similar in older women and men.^17^ The complex relationship between oestrogen and cancer raises questions as to how HRT can be safely provided for women experiencing menopausal symptoms.

Similar to investigations of oestrogen’s role in various cancers, findings from studies of HRT and cancer risk are mixed. For example, HRT use is associated with a reduced risk of liver,^18–20^ oesophageal^20^ and colorectal cancers^20–23^ but an increased risk of glioma and meningioma with use of oestrogen only HRT.^24^ In some cases evidence is conflicting for individual cancers, for example, a recent meta-analysis of lung cancer found a reduced risk in women taking HRT^25^ but an earlier meta-analysis found no effect;^26^ and in non-Hodgkin’s lymphoma, for which a pooled analysis of case-control studies showed decreased incidence in women using HRT,^27^ but two cohort studies found no effect.^28 29^ Furthermore, a cohort study found bladder cancer to be less common in women using combined, but not oestrogen-only HRT,^30^ suggesting that the specific HRT prescription affects its association with cancer.

There are also important questions relating to the risk of HRT use by women who have survived or are living with cancer. This is increasingly important for several reasons: first, improved survival rates from cancer mean female survivors are increasingly likely to experience menopause;^31^ second, in young women with cancer, the associated treatment may cause primary ovarian insufficiency,^31 32^ inducing menopause and thus potentially indicating HRT in this demographic; and third, the induction of ‘surgical’ menopause by hysterectomy and oophorectomy could be an indication for HRT.^33^ While a number of studies have found protective effects of HRT after colorectal cancer,^34–36^ epidemiological evidence at other cancer sites is sparse. For instance, HRT use after cancer diagnosis was associated with reduced mortality in 205 patients with melanoma,^37^ but this was based on only one cancer-specific death in HRT users. Similarly, HRT before diagnosis was associated with reduced mortality in 234 patients with primary liver cancer, but HRT after diagnosis was not investigated.^19^ A case control study of patients with Hodgkin’s lymphoma with and without subsequent breast cancer did not find that risk was increased by HRT.^38^ Finally, in a study of 130 patients with leukaemia no excess recurrences were observed in HRT users.^39^ Further investigation into the potential association between HRT and cancer survival is warranted because existing evidence comes from small studies with limited power, many of which did not investigate HRT after diagnosis, and many cancer sites have not been investigated at all.

Despite the lack of high quality epidemiological evidence, there have been suggestions that HRT should be used cautiously, or not at all, in patients with lung,^31 40^ bladder,^31 41^ gastric^31 41^ and brain cancer.^31 41^ These recommendations may be concerning for patients, and for clinicians there is limited information on providing HRT in women with cancers other than breast cancer in clinical guidelines.^42 43^ This can lead to avoidance of HRT in line with the ‘precautionary principle’, but such an approach has been severely criticised because of the clear benefits of HRT in reducing menopausal symptoms and increasing health-related quality of life.^31 44^ There is a need to clarify the relationship between HRT and non-oestrogen dependent cancers so that women of all ages who survive cancer can make informed choices with their treating clinicians as to the risks and benefits of HRT.

### Risk of cardiovascular disease and venous thromboembolism associated with HRT

In addition to cancer, there are concerns that HRT may lead to cardiovascular complications. A recent Cochrane systematic review with meta-analysis of 22 largely high quality studies found moderate evidence that combined, continuous HRT is associated with an increased risk of myocardial infarction (MI), stroke and venous thromboembolism (VTE), and that oestrogen only, continuous HRT is associated with stroke and VTE but not MI.^8^ The authors concluded that while the absolute risk of these outcomes remained low, the clear association meant that the increased relative risk is a relevant consideration for all women and may contraindicate HRT in those at increased risk of cardiovascular disease (CVD). A more recent systematic review of 33 observational studies, however, had contradictory findings that HRT may protect against CVD, and that the duration, dose, timing and means of HRT administration affected the relationship.^45^ Importantly, the authors noted inconsistent results in the included studies and a wide range in their overall scientific quality. The higher quality of studies included in the Cochrane review lend greater weight to its findings, but the more recent systematic review indicates that further research into the specific type and administration of HRT in high quality studies is needed.

### Summary

There are legitimate concerns about the safety of prescribing HRT for women with cancer and those at elevated risk of VTE or CVD. The role of HRT in improving the quality of life for women during menopause, however, means that understanding how and for whom it can be safely prescribed is essential. Furthermore, the increasing number of women who survive cancer and go on to experience menopausal symptoms suggests that investigation is warranted into this demographic specifically.

### Aims and objectives

Our primary aim is to determine the association between HRT use and the risk of cancer-specific mortality in women with a range of common cancers. Our secondary aim is to quantify the risks of HRT associated with subsequent cancer, all-cause mortality, CVD and VTE in women with cancer. Our study will provide patients with cancer and clinicians with the high quality evidence of the safety of HRT in patients with cancer, allowing them to make informed decisions, and will provide important mechanistic insights into the role of oestrogen in cancer progression.

## Methods and analysis

### Study design and data source

A population-based cohort study will be conducted using the QResearch linked database. QResearch is a large, validated database of anonymised electronic health records and is representative of the English general population.^46^ It compiles patient data at an individual level from GP practices through the Egton Medical Information Systems electronic health records and has linkages to National Health Service (NHS) data through the NHS Digital Hospital Episode Statistics (HES) platform, the English national cancer registry and the English national death register, and has been described in detail previously.^47^

#### Study sample

We will identify an open cohort of women aged 18–79 years who have been registered at a GP clinic contributing data to QResearch for at least 1 year prior to diagnosis of one of the included types of cancer (table 1). Women with cancer will be identified from the English national cancer registry and this will be supplemented with GP clinic records and HES data. The study period is between 1998 and the most recent available data, with censoring at either the date of death or the end of the study period in the primary analysis, and at development of subsequent cancer, CVD or VTE in the relevant secondary analyses. Exclusion criteria are: women with a previous diagnosis of invasive cancer (other than those listed) or who have less than 1 year of follow-up available in the database. Breast cancer will not be included since HRT is contraindicated in breast cancer and oestrogen dependent tumours.^2 42 48^

**Table 1:** Included cancer sites of primary tumours by classification

Category	Specific tumour site
Haematological	Leukaemia, multiple myeloma, non-Hodgkin’s lymphoma
Gastrointestinal	Oral, oesophageal, gastric, liver, pancreatic, colorectal
Gynaecological	Cervical, ovarian, endometrial
Urogenital	Renal, bladder
Other cancer sites	Malignant melanoma, lung, thyroid, brain

#### Exposure

We will extract data on HRT prescriptions from the QResearch database. Treatment will be categorised based on compound (type of oestrogen and type of progestogen where applicable), route of administration (oral, transdermal, vaginal, injection or implant), dose (≤0.625 mg for oral conjugated equine oestrogen, ≤1 mg for oral estradiol, ≤50 µg for transdermal estradiol,^49^ timing (ie, relative to cancer diagnosis) and duration. Daily defined doses (DDDs) will be extrapolated from prescription information where possible; however, it may not be possible in some cases, such as administration by transdermal patch or gel.

### Outcomes

#### Outcomes related to cancer mortality

The primary outcome is cancer specific mortality where cancer is recorded as the primary or underlying cause of death. This will be ascertained using the primary or underlying cause of death as recorded on the linked mortality data from the Office of National Statistics death register. Our secondary outcomes will be incident cases of cancer as recorded on either the GP, hospital, cancer registry or linked mortality record and all-cause mortality.

#### Outcomes related to CVD and VTE

A CVD event will be defined as the earliest record of CVD where it is the primary reason for admission on the GP or HES databases, or where it is recorded as primary cause of death on the mortality record. A CVD event will include coronary heart disease or stroke.

As for CVD, a VTE event will be defined as the earliest record of VTE on any of the three linked data sources (GP, HES or mortality record). VTE will include deep vein thrombosis or pulmonary embolism.

### Confounding variables

Key demographic factors, potentially confounding factors and data relating to the secondary outcome (CVD) are listed below and will be obtained from GP records. Categories of confounders that may apply to both outcomes but with different specific inclusions (eg, pharmacotherapy) are listed in table 2, with specific inclusions shown separately for each outcome.

**Table 2:** Categories of confounders with specific inclusions for each outcome shown separately

	Cancer-related analyses	CVD and VTE-related analyses
Charlson Comorbidity Index (CCI) conditions	Cardiovascular diseases Coronary heart disease (myocardial infarction or angina) Congestive cardiac failure Peripheral vascular disease Stroke/ transient ischaemic attack Gastrointestinal conditions Peptic ulcer disease Liver disease Inflammatory bowel disease Other CCI conditions Dementia Chronic obstructive pulmonary disease Connective tissue disease Haemiplegia Moderate or severe chronic kidney disease Diabetes mellitus (type 1 or type 2) HIV/ AIDS	Gastrointestinal conditions Peptic ulcer disease Liver disease Inflammatory bowel disease Other CCI conditions Dementia Chronic obstructive pulmonary disease Connective tissue disease Hemiplegia Moderate or severe chronic kidney disease Diabetes mellitus (type 1 or type 2) HIV/ AIDS
Other comorbidities	Severe mental illness Depression Venous thromboembolism Vertebrobasilar insufficiency syndrome Anaemia	Severe mental illness Depression Anaemia
Pharmacotherapy (other than HRT and chemotherapy)	Aspirin Statins Losartan Metformin	Anticoagulant drugs—warfarin, oral and parenteral anticoagulants Antihypertensive drugs—Angiotensin Converting Enzyme (ACE)-inhibitors, beta-blockers, calcium channel blockers, diuretics Antiplatelet drugs—aspirin, clopidogrel, dipyridamole, prasugrel, ticagrelor, ticlopidine Mental health drugs Antidepressants—tricyclic antidepressants, compound antidepressants, mono-amine oxidase inhibitors Antipsychotic drugs Analgesic drugs Non-steroidal anti-inflammatories Opioids Drugs of cancer treatment—tamoxifen, aromatase inhibitors, thalidomide, lenalidomide, bevacizumab Immunosuppressant drugs Antidiabetes drugs—insulin, oral hypoglycaemics Prostaglandin analogues Other hormone therapies Oral contraceptive drugs—dianette Topical hormone drugs Megestrol acetate

CVD, cardiovascular disease; HRT, hormone replacement therapy; VTE, venous thromboembolism.

### Demographic and lifestyle factors

- Year of birth.
- Age at cancer diagnosis.
- Townsend deprivation score.
- Most recent body mass index (BMI) prior to outcome diagnosis.
- Family history of cancer.
- Ethnicity (White, Indian, Bangladeshi, Pakistani, Chinese, other Asian, Caribbean, Black African, other).
- Most recent smoking status (never, former, light, moderate, heavy) prior to outcome diagnosis.
- Most recent alcohol consumption status (non-drinker, trivial, light, moderate, heavy, very heavy) prior to outcome diagnosis.
- Geographical region (East of England, East Midlands, West Midlands, London, North East, North West, South Central, South East, South West, Yorkshire and the Humber).^50^

### Tumour-specific factors

#### Histology

- Stage: 1–4 as per Tumour Nodes Metastases classification, or International Federation of Gynaecology and Obstetrics (FIGO) staging system for gynaecological cancers.
- Grade: well, moderately, poorly or not differentiated.

**Cancer treatment within 6 months of diagnosis**^51^

- Radiotherapy.
- Chemotherapy.
- Hormonal therapy for cancer as recorded on the cancer registry.
- Cancer-related surgery:
  - Lung—resection, for example, pneumonectomy or lobectomy.
  - Colorectal—haemicolectomy, colectomy, endoscopic resection.
  - Pancreatic—pancreaticoduodenectomy, distal pancreatectomy.
  - Gynaecological—hysterectomy with or without bilateral salpingo-oophorectomy, pelvic exenterations, cone biopsy or Large Loop Excistion of the Transformation Zone (LLETZ).
  - Gastro-oesophageal—oesophagectomy, gastrectomy.
  - Oral—lip resection, excision of oral lesion, glossectomy, maxillectomy, mandibulectomy, laryngectomy.
  - Thyroid cancers—thyroidectomy with or without neck dissection.
  - Urogenital—nephrectomy, nephroureterectomy, cystectomy.
  - Liver cancers—segmental resection, lobectomy, transplantation.
  - Brain cancers—craniectomy with resection.

#### Other surgery (ie, not cancer related)

- Hysterectomy with or without oophorectomy.

### Statistical analysis

#### Analysis of HRT use after cancer diagnosis

Patients will be followed from 6 months after cancer diagnosis to cancer-specific mortality (censored on death from other causes or end of follow-up). A delay such as this is recommended to exclude deaths related to severe pathology to which HRT use is unlikely to have contributed. The length of the delay will be varied for certain cancers (such as melanoma, non-Hodgkin’s lymphoma, bladder, colorectal and thyroid cancer) as they have better prognosis.

A dose–response analysis will be conducted based on DDD’s, and number and duration of prescriptions. To avoid immortal time bias, HRT use will be modelled as a time varying covariate, that is, patients will only be considered users after a lag of 6 months from their first HRT prescription. Therefore, individuals will be considered users of 0–1 year from 6 months after their first prescription to 6 months after their 12th prescription (or 365th DDD) and >1 year users after this time (table 3A). Other elements of HRT prescription, namely dose, means of administration and whether administration is continuous or cyclical will be investigated through stratification of the sample.

**Table 3:** A–C: Categorisation of hormone replacement therapy (HRT) users for each proposed analysis

Label	Definition
**3A**. HRT use *after* cancer diagnosis	
Non-user	No HRT prescription, or <6 months from first HRT prescription
0–1 year user	6 months after first prescription until 6 months after 12th prescription or 6 months after 365th DDD
>1 year user	Prescriptions beyond above time points
**3B**. HRT use *before* cancer diagnosis	
Non-user	No HRT prescription in the period from 18 months to 6 months before cancer diagnosis
User	HRT prescription in the period from 18 months to 6 months before cancer diagnosis
**3C**. HRT use and CVD	
Non-user	A woman without an HRT prescription
Current user	A woman with a valid HRT prescription
Past user	A woman whose HRT prescription has expired and who does not renew the prescription within 30 days

CVD, cardiovascular disease; DDD, daily defined dose.

Time-dependent Cox regression models will be used to calculate HRs, and 95% CIs, for HRT use after cancer diagnosis adjusting for potential confounders including: age at diagnosis, BMI, year of diagnosis, alcohol and smoking status, ethnicity, deprivation index, stage, grade, cancer treatment, comorbidities, routine medications and prior hysterectomy/oophorectomy.

#### Analysis of HRT use before cancer diagnosis

Patients will be followed from diagnosis to cancer-specific mortality. HRT use will be identified in the 18 months prior to diagnosis, excluding the 6 months immediately prior to diagnosis (table 3B). Exclusion of HRT use in this period is intended to avoid bias arising from potentially short-term prescriptions issued in the lead up to cancer diagnosis that are unlikely to affect the outcome and may result in misclassification.^52^ Cox regression models will be used to calculate HRs and 95% CIs for cancer-specific death in HRT users compared with non-users. Adjusted Cox regression models will include: age at diagnosis, BMI, year of diagnosis, alcohol and smoking status, ethnicity, deprivation index, comorbidities, prior hysterectomy/oophorectomy and routine medication use before diagnosis. Stage, grade and cancer treatments will initially be excluded from the models investigating HRT before diagnosis, as they could lie on the causal pathway. Subsequent analysis, however, will explore their potential role as confounders or effect mediators. Similar analyses will be conducted for secondary outcomes.

#### Confounding factors for specific cancer sites

Analyses will be stratified by cancer site, and some sites will be adjusted for additional covariates that have been shown to be associated with outcome at those sites. For instance, analysis of lung cancer will additionally contain histology (small-cell/non-small-cell) and beta-blocker use; colorectal will additionally contain site (colon, rectum or rectosigmoid colon), family history of colorectal cancer and inflammatory bowel disease; and oesophageal will contain histology (adenocarcinoma and squamous cell).

#### Analysis of CVD

In the secondary analysis of CVD, a start/stop time-varying covariate analysis will be conducted to investigate current HRT use, in which patients will become HRT users on the date of each HRT prescription, and remain HRT users until the end of their current prescription, at which point they will become past HRT users (table 3C) unless they initiate another HRT treatment within 30 days.

Cox regression models will be used to calculate HRs and 95% CIs for CVD events in HRT users and non-users. Adjustment will be made for: age at diagnosis, BMI, ethnicity, alcohol and smoking status, year of diagnosis, comorbidities and medications.

#### Meta-analysis

The HRs and SEs from these analyses will be pooled with identical analyses conducted on cohorts from Scotland and Wales. This two-stage analysis procedure will use random effects models to pool results across cohorts.

### Sensitivity analyses

Confounding will primarily be addressed by entering a wide range of covariates into the Cox regression models. A number of additional sensitivity analyses will also be conducted to check for potential bias and subgroup effects including the following:

1. Analyses will be stratified into age categories of: 18 to <40, 40 to <55 and ≥55 years old. Women younger than 40 are more likely to have experience premature ovarian insufficiency whereas those 55 or over are likely to have experienced natural menopause.
2. An analysis of HRT after cancer diagnosis will be conducted using a new-user design, that is, restricting analyses to patients with cancer who did not use HRT before diagnosis.
3. A negative control analysis will be conducted to check for unmeasured confounding by repeating the main analysis with death from other causes as the outcome because HRT is not expected to influence death from other causes (excluding cancer and CVD).
4. It is possible that poor compliance may confound the analyses. To investigate this an analysis excluding individuals with only one prescription will be conducted, as patients continuing to be prescribed HRT are more likely to be adhering to their prescriptions.
5. A propensity score matched analysis will be conducted. First, logistic regression will be used to determine the propensity to be prescribed HRT. Matching will be implemented in Stata (Statacorp LLC, Texas, USA) to identify a HRT user group and a propensity score matched non-user group. Cox regression will then be used to determine the time to cancer-specific death in the two groups.

### Missing data

Multiple imputation will be used to impute missing values for variables including BMI, smoking and alcohol status, stage and grade according to published guidelines.^53^ As recommended, cancer-specific death status and cumulative hazard will be included in imputation models as will all variables included in regression models. The number of imputed datasets will correspond to the proportion of missing data and results will be combined using Rubin’s rules.^54^

### Limitations

The completeness of stage data will be of importance to the analysis of HRT after diagnosis. Stage completeness is expected to be high for the majority of cancers we propose investigating including lung cancer (~90%); colorectal cancer (~90%) and malignant melanoma (~80%).^55^ For other sites and earlier years of diagnosis, however, stage is more likely to be missing, so it will be imputed by multiple imputation where necessary. Stage completeness is not expected to unduly influence the analysis of HRT before cancer diagnosis on survival and is arguably not appropriate, as it could lie on the causal pathway in these cases.

Smoking and BMI are well recorded in GP records but are not complete. Completeness for smoking is estimated to be approximately 95% in recent UK GP records and for BMI it is roughly 80%. Multiple imputation will be used to impute missing values for these factors.

There are several potential sources of bias that arise from a likely difference in prescribing patterns of HRT for women with cancer. An allocation bias may arise because women with more severe cancer are less likely to be prescribed HRT and therefore more likely to be allocated to the unexposed group. Alternatively, there may be a general aversion to prescribing HRT in this population, so that only those women whose cancers have a high chance of remission are considered for HRT. We will seek to address this in several ways, first, these concerns would not affect prescription patterns prior to cancer diagnosis, second, a ‘new-user’ sensitivity analysis will be conducted which excludes individuals with a history of HRT use prior to their cancer diagnosis. These analyses will provide a basis for assessing the potential impact of differing prescribing patterns and allocation bias.

### Sample size calculation

Detectable HRs were calculated based on case numbers from the English study cohort (table 4). Statistical power to detect a difference (the beta value) was set at 90% and significance (the alpha value) at 5%. We estimated that 7% of female patients with cancer would use HRT after diagnosis, consistent with published reports of roughly 5% HRT usage in a Swedish population-based colorectal cancer cohort after diagnosis,^34^ and 9% HRT usage in a Danish melanoma cohort after diagnosis.^56^ These calculations show that we would be able to detect HRs of 1.5 or greater with HRT use after diagnosis for most cancer sites.

**Table 4:** Detectable HRs for each type of cancer assuming 90% power to detect a difference and statistical significance at p<0.05

Cancer type	Total cases	Cancer specific mortality				All-cause mortality			
		HRT prior to diagnosis*		HRT postdiagnosis analysis†		HRT prior to diagnosis*		HRT postdiagnosis analysis†	
		Total deaths	Detectable HR	Deaths >6 months from diagnosis	Detectable HR	Total deaths	Detectable HR	Deaths >6 months from diagnosis	Detectable HR
Gastrointestinal									
Oral	5855	901	1.53 (or 0.65)	649	1.65 (or 0.61)	2350	1.30 (or 0.74)	1762	1.35 (or 0.74)
Oesophageal	11 462	4077	1.22 (or 0.82)	2237	1.37 (or 0.73)	8591	1.15 (or 0.87)	4415	1.21 (or 0.83)
Gastric	5579	2735	1.28 (or 0.78)	1241	1.44 (or 0.70)	4134	1,22 (or 0.82)	2002	1.32 (or 0.75)
Pancreatic	9940	7110	1.16 (or 0.86)	2263	1.31 (or 0.77)	8320	1.15 (or 0.87)	2683	1.28 (or 0.78)
Liver	3984	2405	1.30 (or 0.77)	813	1.57 (or 0.64)	3182	1,25 (or 0.80)	1068	1.48 (or 0.68)
Colorectal	37 915	10 265	1.13 (or 0.88)	6260	1.17 (or 0.85)	17 989	1.10 (or 0.91)	11 418	1.13 (or 0.89)
Other									
Lung	40 084	24 904	1.08 (or 0.92)	10 582	1.13 (or 0.88)	30 450	1.08 (or 0.93)	13 250	1.12 (or 0.90)
Melanoma	16 382	1059	1.48 (or 0.68)	902	1.53 (or 0.65)	2558	1.29 (or 0.78)	2277	1.31 (or 0.77)
Thyroid	3876	321	2.04 (or 0.49)	143	2.89 (or 0.35)	554	1.72 (or 0.58)	325	2.04 (or 0.49)
Brain	4759	2422	1.29 (or 0.77)	1059	1.48 (or 0.68)	3319	1.25 (or 0.80)	1362	1.41 (or 0.71)
Haematological									
Leukaemia	8620	2593	1.28 (or 0.78)	1258	1.43 (or 0.70)	4326	1.21 (or 0.82)	2494	1.29 (or 0.78)
Multiple myeloma	5381	1753	1.36 (or 0.74)	1251	1.43 (or 0.70)	2842	1.27 (or 0.79)	2022	1.33 (or 0.75)
Non-Hodgkin’s lymphoma	12 584	2920	1.26 (or 0.79)	1665	1.37 (or 0.73)	5166	1.19 (or 0.84)	3218	1.25 (or 0.80)
Gynaecological									
Cervical	5594	1093	1.47 (or 0.68)	784	1.57 (or 0.63)	1753	1.36 (or 0.74)	1265	1.43 (or 0.70)
Endometrial	18 855	2913	1.27 (or 0.79)	2188	1.31 (or 0.76)	5821	1.18 (or 0.85)	4557	1.21 (or 0.83)
Ovarian	16 526	6444	1.17 (or 0.85)	2153	1.22 (or 0.82)	8953	1.14 (or 0.87)	5793	1.18 (or 0.85)
Urinary									
Renal	7895	2153	1.32 (or 0.76)	1167	1.45 (or 0.69)	3537	1.24 (or 0.81)	2124	1.32 (or 0.76)
Bladder	10 507	2256	1.31 (or 0.76)	1301	1.42 (or 0.70)	4440	1.21 (or 0.83)	3068	1.26 (or 0.79)

*Uses total number of deaths.

† Uses number of deaths more than 6 months after diagnosis only.

HRT, hormone replacement therapy.

## Ethics and dissemination

Ethical approval for this project was obtained from the QResearch scientific committee (Ref: OX24, project title ‘Use of hormone replacement therapy and survival from cancer’). Ethical approval for the QResearch database is obtained annually from East Midlands - Derby Research Ethics Committee (Ref: 18/EM/0400).

The findings of this study will be submitted for peer-reviewed publication in an academic journal. In addition, the findings will be provided to Cancer Research UK for public dissemination, with the assistance of the Patient and Public Involvement (PPI) panel.

## Patient and public involvement

The project will be supported throughout by a PPI panel, made up of women with experience of non-oestrogenic cancers. Members of the Northern Ireland Cancer Research Consumer Forum, patients with a cancer group, provided feedback on the project proposal before submission to Cancer Research United Kingdom (CRUK).

Another PPI panel of four women with a previous diagnosis of cancer was convened to inform the English component of this project (ie, the one presented in this protocol). They agreed the study was important and potentially valuable for female cancer survivors who experience menopausal symptoms. The panel suggested that both cancer-specific mortality and subsequent cancer diagnoses would be of interest to women in this demographic considering whether to use HRT. Future discussions will seek feedback on the relevance and resonance of findings and guide us on research communications and dissemination.

## Supplementary Material

Reviewer comments

Author's manuscript
