# Supplementary material for: Hormone replacement therapy and cancer survival: a longitudinal cohort study: protocol paper
Source: BMJ Open. 2021 Aug 2;11(8):e046701. doi: 10.1136/bmjopen-2020-046701 (PMC8330592; doi:10.1136/bmjopen-2020-046701)
Supplement: Author's manuscript [file bmjopen-2020-046701.draft_revisions.pdf]

BMJ Open

BMJ Open is committed to open peer review. As part of this commitment we make the peer review history of every article we publish publicly available.

When an article is published we post the peer reviewers' comments and the authors' responses online. We also post the versions of the paper that were used during peer review. These are the versions that the peer review comments apply to.

The versions of the paper that follow are the versions that were submitted during the peer review process. They are not the versions of record or the final published versions. They should not be cited or distributed as the published version of this manuscript.

BMJ Open is an open access journal and the full, final, typeset and author-corrected version of record of the manuscript is available on our site with no access controls, subscription charges or pay-per-view fees (http://bmjopen.bmj.com).

If you have any questions on BMJ Open's open peer review process please email info.bmjopen@bmj.com

BMJ Open

Hormone Replacement Therapy and Cancer Survival: A Longitudinal Cohort Study - protocol paper

Journal:	*BMJ Open*
Manuscript ID	bmjopen-2020-046701
Article Type:	Protocol
Date Submitted by the Author:	06-Nov-2020
Complete List of Authors:	Ranger, Tom; Oxford University, Nuffield Department of Primary Care Health Sciences<br>Burchardt, Judith; Oxford University, Nuffield Department of Primary Care Health Sciences<br>Clift, Ashley; University of Oxford, Nuffield Department of Primary Care Health Sciences<br>Mei, Xue; Oxford University, Nuffield Department of Primary Care Health Sciences<br>Tan, Pui San; University of Oxford, Nuffield Department of Primary Care Health Sciences<br>Coupland, Carol; University of Nottingham, Division of Primary Care<br>Dixon, Sharon; University of Oxford, Nuffield Department of Primary Care Health Sciences<br>Cardwell, Christopher; Queen's University Belfast, School of Medicine, Dentistry and Biomedical Sciences<br>Hippisley-Cox, Julia; University of Oxford, Nuffield Department of Primary Care Sciences
Keywords:	EPIDEMIOLOGY, ONCOLOGY, Sex steroids & HRT < DIABETES & ENDOCRINOLOGY, CARDIOLOGY

SCHOLARONE™
Manuscripts

I, the Submitting Author has the right to grant and does grant on behalf of all authors of the Work (as defined in the below author licence), an exclusive licence and/or a non-exclusive licence for contributions from authors who are: i) UK Crown employees; ii) where BMJ has agreed a CC-BY licence shall apply, and/or iii) in accordance with the terms applicable for US Federal Government officers or employees acting as part of their official duties; on a worldwide, perpetual, irrevocable, royalty-free basis to BMJ Publishing Group Ltd ("BMJ") its licensees and where the relevant Journal is co-owned by BMJ to the co-owners of the Journal, to publish the Work in this journal and any other BMJ products and to exploit all rights, as set out in our licence.

The Submitting Author accepts and understands that any supply made under these terms is made by BMJ to the Submitting Author unless you are acting as an employee on behalf of your employer or a postgraduate student of an affiliated institution which is paying any applicable article publishing charge ("APC") for Open Access articles. Where the Submitting Author wishes to make the Work available on an Open Access basis (and intends to pay the relevant APC), the terms of reuse of such Open Access shall be governed by a Creative Commons licence – details of these licences and which Creative Commons licence will apply to this Work are set out in our licence referred to above.

Other than as permitted in any relevant BMJ Author's Self Archiving Policies, I confirm this Work has not been accepted for publication elsewhere, is not being considered for publication elsewhere and does not duplicate material already published. I confirm all authors consent to publication of this Work and authorise the granting of this licence.

For peer review only

Hormone Replacement Therapy and Cancer Survival: A Longitudinal Cohort Study - protocol paper

Tom A. Ranger[1], Judith Burchardt[1], Ashley K. Clift[1,2], Winnie Mei[1], Carol Coupland[3], Pui San Tan[1], Sharon Dixon[1], Chris Cardwell[4], Julia Hippisley-Cox[1]

1. Primary Care Epidemiology, Nuffield Department of Primary Care Health Sciences, Medical Sciences Division, University of Oxford, Oxford, UK
2. Cancer Research UK Oxford Centre, Department of Oncology, Old Road Campus Research Building, Roosevelt Drive, Oxford, UK
3. Division of Primary Care, University of Nottingham, Nottingham, UK
4. Cancer Epidemiology and Health Services Research Group (CRC, MMC), Centre for Public Health, Queen's University Belfast, Royal Victoria Hospital Site, Belfast, UK

For peer review only

Running head: HRT and cancer survival

Date and version: November 2020, version 1

Corresponding author:

Dr. Tom Ranger, PhD
Nuffield Department of Primary Care Health Sciences,
Radcliffe Primary Care Building,
University of Oxford,
Oxford, UK

For peer review only - http://bmjopen.bmj.com/site/about/guidelines.xhtml

Abstract

Introduction

Hormone replacement therapy (HRT) can help women experiencing menopausal symptoms, but usage has declined due to uncertainty around risks of cancer and some cardio-vascular diseases (CVD). Moreover, improved cancer survival rates mean that more women who survive cancer go on to experience menopausal symptoms. Understanding these relationships is important so that women and their clinicians can make informed decisions around the risks and benefits of HRT.

This study's primary aim is to determine the association between HRT use after cancer diagnosis and the risk of cancer-specific mortality. The secondary aims are to investigate the risks of HRT on subsequent cancer, all-cause mortality and CVD.

Methods and analysis

We will conduct a population-based longitudinal cohort study of 18 to 79-year-old women diagnosed with cancer between 1998 and 2020, using the QResearch database. The main exposure is HRT use, categorised based on compound, dose and route of administration, and modeled as a time-varying covariate. Analysis of HRT use pre- and post-cancer diagnosis will be conducted separately.

The primary outcome is cancer-specific mortality, which will be stratified by cancer site. Secondary outcomes include subsequent cancer diagnosis, CVD (including venous thrombo-embolism) and all-cause mortality. Adjustment will be made for key confounders such as age, BMI, ethnicity, deprivation index, comorbidities, and cancer grade, stage and treatment. Statistical analysis will include descriptive statistics and Cox proportional hazards models to calculate hazard ratios and 95% confidence intervals.

Ethics and dissemination

Ethical approval for this project was obtained from the QResearch Scientific Committee [Ref: OX24, project title "Use of hormone replacement therapy and survival from cancer"]. This project has been, and will continue to be, supported by patient and public involvement panels. We intend to the submit the findings for peer-reviewed publication in an academic journal and disseminate them to the public through Cancer Research UK.

For peer review only - http://bmjopen.bmj.com/site/about/guidelines.xhtml

Strengths and limitations of this study

- This is an open cohort study comprising a nationally representative sample of English women.
- The cohort consists of GP clinic data linked to hospital records, the English national cancer registry and English national death registry.
- This study has access to detailed information on HRT prescriptions, allowing analysis with consideration of the specific compound, dose, route of administration and duration of exposure.
- This study is limited by high rates of missing data for cancer grade and stage, although completeness has improved in recent years, this will be accounted for using appropriate multiple imputation techniques.

For peer review only

For peer review only - http://bmjopen.bmj.com/site/about/guidelines.xhtml

Background

Prevalence of HRT use

Hormone replacement therapy (HRT) involves clinical provision of the female sex hormone oestrogen either on its own or in combination with another hormone, progestogen. It is an effective and important treatment for symptoms of the menopause, including vasomotor symptoms (hot flushes and night sweats), urogenital atrophy[1] and postmenopausal osteoporosis[2]. Yet between 2002 and 2005, HRT usage in the UK dropped from approximately 29% of postmenopausal women to approximately 11%[3], and this pattern was consistent across Europe[4]. The drop in usage has been attributed to the widely reported findings of the Million Women's Study[5] and the early cessation of the Women's Health Initiative trial[3 4 6], which concluded that an observed increase in risk of cardiovascular events and breast cancer in women taking combined oestrogen and progestogen HRT outweighed the benefits of reduced rates of colorectal cancer and osteoporotic fractures[7]. Evidence from a recent Cochrane systematic review and meta-analysis though, suggests that HRT remains an important treatment option for women with severe menopausal symptoms who do not have other risk factors, such as oestrogen sensitive cancers[8]. Given the demonstrated risks and benefits of HRT, there is a need to clarify which women can use it safely, to treat menopausal symptoms without unduly increasing their risk of adverse outcomes.

HRT in women with cancer

The role of oestrogen in cancer is complex, with evidence that it accelerates cancer progression in some sites but slows it in others. For instance, pre-clinical studies have shown that oestrogen stimulates growth in bladder[9] and gastric cancer cell lines[10] but, in contrast, similar studies found that it inhibits cell growth and/or induces apoptosis in oesophageal adenocarcinoma[11] and malignant melanoma cell lines[12]. What's more, in a mouse model oestrogen increased the number and volume of lung adenocarcinomas[13], and molecular pathological studies have shown oestrogen receptor expression is associated with metastasis and poorer survival in gastric cancer[14]. Epidemiological studies also report contrasting findings depending the cancer of interest, with some showing markedly lower incidence and slightly better survival for women compared to men for many cancers[15 16], whereas another study demonstrated survival after gastric cancer is worse in young women (<45 years old) than in young men, but similar in older women and men[17]. The complex relationship between oestrogen and cancer raises questions as to how HRT can be safely provided for women experiencing menopausal symptoms.

For peer review only - http://bmjopen.bmj.com/site/about/guidelines.xhtml

Similar to investigations of oestrogen's role in various cancers, findings from studies of HRT and cancer risk are mixed. For example, HRT use is associated with a reduced risk of liver[18-20], oesophageal[20] and colorectal cancers[20-23] but an increased risk of glioma and meningioma with use of oestrogen only HRT[24]. In some cases evidence is conflicting for individual cancers, for example, a recent meta-analysis of lung cancer found a reduced risk in women taking HRT[25] but an earlier meta-analysis found no effect[26]; and in non-Hodgkin lymphoma, for which a pooled analysis of case-control studies showed decreased incidence in women using HRT[27], but two cohort studies found no effect[28 29]. Furthermore, a cohort study found bladder cancer to be less common in women using combined, but not oestrogen-only HRT[30], suggesting that the specific HRT prescription affects its association with cancer.

There are also important questions relating to the risk of HRT use by women who have survived or are living with cancer. This is increasingly important for several reasons: first, improved survival rates from cancer mean female survivors are increasingly likely to experience menopause[31]; second, in young women with cancer, the associated treatment may cause primary ovarian insufficiency (POI)[31 32], inducing menopause and thus potentially indicating HRT in this demographic; and third, the induction of 'surgical' menopause by hysterectomy and oophorectomy could be an indication for HRT[33]. While a number of studies have found protective effects of HRT after colorectal cancer[34-36], epidemiological evidence at other cancer sites is sparse. For instance, HRT use after cancer diagnosis was associated with reduced mortality in 205 melanoma patients[37], but this was based upon only one cancer-specific death in HRT users. Similarly, HRT before diagnosis was associated with reduced mortality in 234 patients with primary liver cancer, but HRT after diagnosis was not investigated[19]. A case control study of Hodgkin's lymphoma patients with and without subsequent breast cancer did not find that risk was increased by HRT[38]. Finally, in a study of 130 leukaemia patients no excess recurrences were observed in HRT users[39]. Further investigation into the potential association between HRT and cancer survival is warranted because existing evidence comes from small studies with limited power, many of which did not investigate HRT after diagnosis, and many cancer sites have not been investigated at all.

Despite the lack of high quality epidemiological evidence there have been suggestions that HRT should be used cautiously, or not at all, in patients with lung[31 40], bladder[31 41], gastric[31 41], and brain cancer[31 41]. These recommendations may be concerning for patients, and for clinicians there is limited information on providing HRT in women with cancers other than breast cancer in clinical guidelines[42 43]. This can lead to avoidance of HRT in line with the 'precautionary principle', but such an approach has been severely criticized because of the clear benefits of HRT in reducing menopausal symptoms and increasing health

For peer review only - http://bmjopen.bmj.com/site/about/guidelines.xhtml

related quality of life[31 44]. There is a need to clarify the relationship between HRT and non-oestrogen dependent cancers so that women of all ages who survive cancer can make informed choices with their treating clinicians as to the risks and benefits of HRT.

Risk of cardiovascular disease and venous thrombo-embolism associated with HRT

In addition to cancer, there are concerns that HRT may lead to cardiovascular complications. A recent Cochrane systematic review with meta-analysis of 22 largely high quality studies found moderate evidence that combined, continuous HRT is associated with an increased risk of myocardial infarction (MI), stroke and venous thromboembolism (VTE), and that oestrogen only, continuous HRT is associated with stroke and VTE but not MI[8]. The authors concluded that while the absolute risk of these outcomes remained low, the clear association meant that the increased relative risk is a relevant consideration for all women and may contraindicate HRT in those at increased risk of cardiovascular disease (CVD). A more recent systematic review of 33 observational studies, however, had contradictory findings that HRT may protect against CVD, and that the duration, dose, timing and means of HRT administration affected the relationship[45]. Importantly, the authors noted inconsistent results in the included studies and a wide range in their overall scientific quality. The higher quality of studies included in the Cochrane review lend greater weight to its findings, but the more recent systematic review indicates that further research into the specific type and administration of HRT in high quality studies is needed.

Summary

There are legitimate concerns about the safety of prescribing HRT for women with cancer and those at elevated risk of VTE or CVD. The role of HRT in improving the quality of life for women during menopause, however, means that understanding how and for whom it can be safely prescribed is essential. Furthermore, the increasing number of women who survive cancer and go on to experience menopausal symptoms suggests that investigation is warranted into this demographic specifically.

Aims and objectives

For peer review only - http://bmjopen.bmj.com/site/about/guidelines.xhtml

Our primary aim is to determine the association between HRT use and the risk of cancer-specific mortality in women with a range of common cancers. Our secondary aim is to quantify the risks of HRT associated with subsequent cancer, all-cause mortality, CVD and VTE in women with cancer. Our study will provide cancer patients and clinicians with the high quality evidence of the safety of HRT in cancer patients, allowing them to make informed decisions, and will provide important mechanistic insights into the role of oestrogen in cancer progression.

Methods and analysis

Study design and data source

A population-based cohort study will be conducted using the QResearch linked database. QResearch is a large, validated database of anonymized electronic health records and is representative of the English general population[46]. It compiles patient data at an individual level from GP practices through the Egton Medical Information Systems (EMIS) electronic health records and has linkages to the NHS Digital Hospital Episode Statistics (HES) platform, the English national cancer registry and the English national death register, and has been described in detail previously[47].

Study sample

We will identify an open cohort of women aged 18 to 79 years who have been registered at a GP clinic contributing data to QResearch for at least 1 year prior to diagnosis of one of the included types of cancer (Table 1). Women with cancer will be identified from the English national cancer registry and this will be supplemented with GP clinic records and HES data. The study period is between 1998 and the most recent available data, with censoring at either the date of death or the end of the study period in the primary analysis, and at development of subsequent cancer, CVD or VTE in the relevant secondary analyses. Exclusion criteria are: women with a previous diagnosis of invasive cancer (other than those listed) or who have less than one year of follow-up available in the database. Breast cancer will not be included since HRT is contraindicated in breast cancer and oestrogen dependent tumours[2 42 48].

Table 1. Included cancer sites of primary tumours by classification

For peer review only - http://bmjopen.bmj.com/site/about/guidelines.xhtml

Category	Specific Tumour Site
Haematological	Leukaemia, multiple myeloma, non-Hodgkin's lymphoma
Gastro-intestinal	Oral, oesophageal, gastric, liver, pancreatic, colorectal
Gynaecological	Cervical, ovarian, endometrial
Urogenital	Renal, bladder
Other cancer sites	Malignant melanoma, lung, thyroid, brain

Exposure

We will extract data on HRT prescriptions from the QResearch database. Treatment will be categorized based on compound (type of oestrogen and type of progestogen where applicable), route of administration (oral, transdermal, vaginal, injection or implant), dose (≤0.625 mg for oral conjugated equine oestrogen, ≤1 mg for oral estradiol, ≤50 μg for transdermal estradiol[49]), timing (i.e. relative to cancer diagnosis) and duration. Daily defined doses (DDDs) will be extrapolated from prescription information where possible, however, it may not be possible in some cases, such as administration by transdermal patch or gel.

Outcomes

Outcomes related to cancer mortality

The primary outcome is cancer specific mortality where cancer is recorded as the primary or underlying cause of death. This will be ascertained using the primary or underlying cause of death as recorded on the linked mortality data from the Office of National Statistics death register. Our secondary outcomes will be incident cases of cancer as recorded on either the GP, hospital, cancer registry or linked mortality record, and all-cause mortality.

Outcomes related to CVD and VTE

A CVD event will be defined as the earliest record of CVD where it is the primary reason for admission on the GP or HES databases, or where it is recorded as primary cause of death on the mortality record. A CVD event will include coronary heart disease or stroke.

For peer review only

As for CVD, a VTE event will be defined as the earliest record of VTE on any of the three linked data sources (GP, hospital episode statistics or mortality record). VTE will include deep vein thrombosis or pulmonary embolism.

Confounding variables

Key demographic factors, potentially confounding factors and data relating to the secondary outcome (CVD) are listed below and will be obtained from GP records. Categories of confounders that may apply to both outcomes but with different specific inclusions (e.g. pharmacotherapy) are listed in Table 2, with specific inclusions shown separately for each outcome.

Demographic and lifestyle factors

- Year of birth
- Age at cancer diagnosis
- Townsend deprivation score
- Most recent Body Mass Index (BMI) prior to outcome diagnosis
- Family history of cancer
- Ethnicity (White, Indian, Bangladeshi, Pakistani, Chinese, other Asian, Caribbean, Black African, other)
- Most recent smoking status (never, former, light, moderate, heavy) prior to outcome diagnosis
- Most recent alcohol consumption status (non-drinker, trivial, light, moderate, heavy, very heavy) prior to outcome diagnosis
- Geographical region (East of England, East Midlands, West Midlands, London, North East, North West, South Central, South East, South West, Yorkshire and the Humber)[50]

For peer review only - http://bmjopen.bmj.com/site/about/guidelines.xhtml

Table 2. Categories of confounders with specific inclusions for each outcome shown separately

	**Cancer related analyses**	**CVD and VTE related analyses**
Charlson Comorbidity Index (CCI) conditions	Cardiovascular diseases<br>• Coronary heart disease (myocardial infarction or angina)<br>• Congestive cardiac failure<br>• Peripheral vascular disease<br>• Stroke/ transient ischaemic attack<br>Gastro-intestinal conditions<br>• Peptic ulcer disease<br>• Liver disease<br>• Inflammatory bowel disease<br>Other CCI conditions<br>• Dementia<br>• Chronic obstructive pulmonary disease<br>• Connective tissue disease<br>• Hemiplegia<br>• Moderate or severe chronic kidney disease<br>• Diabetes mellitus (type 1 or type 2)<br>• HIV/ AIDS	Gastro-intestinal conditions<br>• Peptic ulcer disease<br>• Liver disease<br>• Inflammatory bowel disease<br>Other CCI conditions<br>• Dementia<br>• Chronic obstructive pulmonary disease<br>• Connective tissue disease<br>• Hemiplegia<br>• Moderate or severe chronic kidney disease<br>• Diabetes mellitus (type 1 or type 2)<br>• HIV/ AIDS
Other comorbidities	• Severe mental illness	• Severe mental illness

For peer review only

	• Depression<br>• Venous thromboembolism<br>• VBI syndrome<br>• Anaemia	• Depression<br>• Anaemia
Pharmacotherapy (other than HRT and chemotherapy)	• Aspirin<br>• Statins<br>• Losartan<br>• Metformin	• Anticoagulant drugs - warfarin, oral and parenteral anticoagulants<br>• Antihypertensive drugs – ACE-inhibitors, beta-blockers, calcium channel blockers, diuretics<br>• Antiplatelet drugs – aspirin, clopidogrel, dipyridamole, prasugrel, ticagrelor, ticlopidine<br>• Mental health drugs<br>○ Antidepressants - tricyclic antidepressants, compound antidepressants, mono-amine oxidase inhibitors<br>○ Antipsychotic drugs<br>• Analgesic drugs<br>○ Non-steroidal anti-inflammatories<br>○ Opioids<br>• Drugs of cancer treatment - tamoxifen, aromatase inhibitors, thalidomide, lenalidomide, bevacizumab

For peer review only - http://bmjopen.bmj.com/site/about/guidelines.xhtml

		• Immunosuppressant drugs<br>• Antidiabetes drugs - insulin, oral hypoglycaemics<br>• Prostaglandin analogues<br>• Other hormone therapies<br>○ Oral contraceptive drugs - dianette<br>○ Topical hormone drugs<br>○ Megestrol acetate

For peer review only

For peer review only - http://bmjopen.bmj.com/site/about/guidelines.xhtml

Tumour specific factors

Histology

- Stage: 1-4 as per Tumour Nodes Metastases classification, or FIGO for gynaecological cancers
- Grade: well, moderately, poorly or not differentiated

Cancer treatment within 6 months of diagnosis[51]

- Radiotherapy
- Chemotherapy
- Hormonal therapy for cancer as recorded on the cancer registry
- Cancer related surgery
  - Lung – resection, e.g. pneumonectomy or lobectomy
  - Colorectal – hemicolectomy, colectomy, endoscopic resection
  - Pancreatic – pancreaticoduodenectomy, distal pancreatectomy
  - Gynaecological – hysterectomy with or without bilateral salpingo-oophorectomy, pelvic exenterations, cone biopsy or LLTEZ
  - Gastro-oesophageal – oesophagectomy, gastrectomy
  - Oral – lip resection, excision of oral lesion, glossectomy, maxillectomy, mandibulectomy, laryngectomy
  - Thyroid cancers – thyroidectomy with or without neck dissection
  - Urogenital – nephrectomy, nephroureterectomy, cystectomy
  - Liver cancers – segmental resection, lobectomy, transplantation
  - Brain cancers – craniectomy with resection

For peer review only

Other surgery (i.e. not cancer related)

- Hysterectomy with or without oophorectomy

Statistical analysis

Analysis of HRT use after cancer diagnosis

Patients will be followed from 6 months after cancer diagnosis to cancer-specific mortality (censored on death from other causes or end of follow-up). A delay such as this is recommended to exclude deaths

For peer review only - http://bmjopen.bmj.com/site/about/guidelines.xhtml

related to severe pathology to which HRT use is unlikely to have contributed. The length of the delay will be varied for certain cancers (such as melanoma, non-Hodgkin's lymphoma, bladder, colorectal and thyroid cancer) as they have better prognosis.

A dose-response analysis will be conducted based upon DDD's, and number and duration of prescriptions. To avoid immortal time bias, HRT use will be modelled as a time varying covariate, i.e. patients will only be considered users after a lag of 6 months from their first HRT prescription. Therefore, individuals will be considered users of 0 to 1 year from 6 months after their first prescription to 6 months after their 12th prescription (or 365th DDD) and >1 year users after this time (Table 3A). Other elements of HRT prescription, namely dose, means of administration and whether administration is continuous or cyclical will be investigated through stratification of the sample.

Time-dependent Cox regression models will be used to calculate Hazard Ratios (HRs), and 95% Confidence Intervals (CIs), for HRT use after cancer diagnosis adjusting for potential confounders including: age at diagnosis, BMI, year of diagnosis, alcohol and smoking status, ethnicity, deprivation index, stage, grade, cancer treatment, comorbidities, routine medications and prior hysterectomy/ oophorectomy.

Analysis of HRT use before cancer diagnosis

Patients will be followed from diagnosis to cancer-specific mortality. HRT use will be identified in the 18 months prior to diagnosis, excluding the 6 months immediately prior to diagnosis (Table 3B). Exclusion of HRT use in this period is intended to avoid bias arising from potentially short-term prescriptions issued in the lead up to cancer diagnosis that are unlikely to affect the outcome and may result in misclassification[52]. Cox regression models will be used to calculate HRs and 95% CIs for cancer-specific death in HRT users compared with non-users. Adjusted Cox regression models will include: age at diagnosis, BMI, year of diagnosis, alcohol and smoking status, ethnicity, deprivation index, comorbidities, prior hysterectomy/oophorectomy and routine medication use before diagnosis. Stage, grade and cancer treatments will initially be excluded from the models investigating HRT before diagnosis, as they could lie on the causal pathway. Subsequent analysis, however, will explore their potential role as confounders or effect mediators. Similar analyses will be conducted for secondary outcomes.

For peer review only - http://bmjopen.bmj.com/site/about/guidelines.xhtml

Confounding factors for specific cancer sites

Analyses will be stratified by cancer site, and some sites will be adjusted for additional covariates that have been shown to be associated with outcome at those sites. For instance, analysis of lung cancer will additionally contain histology (small-cell/non-small-cell) and beta-blocker use; colorectal will additionally contain site (colon, rectum or rectosigmoid colon), family history of colorectal cancer and inflammatory bowel disease; and oesophageal will contain histology (adenocarcinoma and squamous cell).

Analysis of CVD

In the secondary analysis of cardiovascular disease, a start/ stop time-varying covariate analysis will be conducted to investigate current HRT use, in which patients will become HRT users on the date of each HRT prescription, and remain HRT users until the end of their current prescription, at which point they will become past HRT users (Table 3C) unless they initiate another HRT treatment within 30 days.

Cox regression models will be used to calculate HRs and 95%CIs for CVD events in HRT users and non-users. Adjustment will be made for: age at diagnosis, BMI, ethnicity, alcohol and smoking status, year of diagnosis, comorbidities and medications.

Table 3A-C. Categorisation of HRT users for each proposed analysis

**Label**	**Definition**
**3A.** HRT use *after* cancer diagnosis	
Non-user	No HRT prescription, or <6 months from first HRT prescription
0-1 year user	6 months after 1st prescription until 6 months after 12th prescription or 6 months after 365th DDD
>1 year user	Prescriptions beyond above time points
**3B.** HRT use *before* cancer diagnosis	
Non-user	No HRT prescription in the period from 18 months to 6 months before cancer diagnosis
User	HRT prescription in the period from 18 months to 6 months before cancer diagnosis
**3C.** HRT use and CVD	
Non-user	A woman without an HRT prescription
Current user	A woman with a valid HRT prescription

For peer review only - http://bmjopen.bmj.com/site/about/guidelines.xhtml

Past user	A woman whose HRT prescription has expired and who does not renew the prescription within 30 days

HRT – hormone replacement therapy; CVD – cardiovascular disease

Meta-analysis

The HRs and Standard Errors (SEs) from these analyses will be pooled with identical analyses conducted on cohorts from Scotland and Wales. This two-stage analysis procedure will use random effects models to pool results across cohorts.

Sensitivity analyses

Confounding will primarily be addressed by entering a wide range of covariates into the Cox regression models. A number of additional sensitivity analyses will also be conducted to check for potential bias and sub-group effects including the following:

1. Analyses will be stratified into age categories of: 18 to <40, 40 to <55 and ≥55 years old. Women younger than 40 are more likely to have experience premature ovarian insufficiency whereas those 55 or over are likely to have experienced natural menopause.
2. An analysis of HRT after cancer diagnosis will be conducted using a new-user design i.e. restricting analyses to cancer patients who did not use HRT before diagnosis.
3. A negative control analysis will be conducted to check for unmeasured confounding by repeating the main analysis with death from other causes as the outcome because HRT is not expected to influence death from other causes (excluding cancer and cardiovascular disease).
4. It is possible that poor compliance may confound the analyses. To investigate this an analysis excluding individuals with only one prescription will be conducted, as patients continuing to be prescribed HRT are more likely to be adhering to their prescriptions.
5. A propensity score matched analysis will be conducted. First, logistic regression will be used to determine the propensity to be prescribed HRT. Matching will be implemented in Stata (Statacorp LLC, Texas, USA) to identify a HRT user group and a propensity score matched non-user group. Cox regression will then be used to determine the time to cancer-specific death in the two groups.

For peer review only - http://bmjopen.bmj.com/site/about/guidelines.xhtml

Missing data

Multiple imputation will be used to impute missing values for variables including BMI, smoking and alcohol status, stage and grade according to published guidelines[53]. As recommended, cancer-specific death status and cumulative hazard will be included in imputation models as will all variables included in regression models. The number of imputed datasets will correspond to the proportion of missing data and results will be combined using Rubin's rules[54].

Limitations

The completeness of stage data will be of importance to the analysis of HRT after diagnosis. Stage completeness is expected to be high for the majority of cancers we propose investigating including lung cancer (~90%); colorectal cancer (~90%) and malignant melanoma (~80%)[55]. For other sites and earlier years of diagnosis, however, stage is more likely to be missing, so it will be imputed by multiple imputation where necessary. Stage completeness is not expected to unduly influence the analysis of HRT before cancer diagnosis on survival and is arguably not appropriate, as it could lie on the causal pathway in these cases.

Smoking and BMI are well recorded in GP records but are not complete. Completeness for smoking is estimated to be approximately 95% in recent UK GP records and for BMI it is roughly 80%. Multiple imputation will be used to impute missing values for these factors.

There are several potential sources of bias that arise from a likely difference in prescribing patterns of HRT for women with cancer. An allocation bias may arise because women with more severe cancer are less likely to be prescribed HRT and therefore more likely to be allocated to the unexposed group. Alternatively, there may be a general aversion to prescribing HRT in this population, so that only those women whose cancers have a high chance of remission are considered for HRT. We will seek to address this in several ways, first, these concerns would not affect prescription patterns prior to cancer diagnosis, second, a 'new-user' sensitivity analysis will be conducted which excludes individuals with a history of HRT use prior to their cancer diagnosis. These analyses will provide a basis for assessing the potential impact of differing prescribing patterns and allocation bias.

Sample size calculation

For peer review only - http://bmjopen.bmj.com/site/about/guidelines.xhtml

Detectable hazard ratios were calculated based on case numbers from the English study cohort (Table 4). Statistical power to detect a difference (the beta value) was set at 90% and significance (the alpha value) at 5%. We estimated that 7% of female cancer patients would use HRT after diagnosis, consistent with published reports of roughly 5% HRT usage in a Swedish population based colorectal cancer cohort after diagnosis[56], and 9% HRT usage in a Danish melanoma cohort after diagnosis[57]. These calculations show that we would be able to detect HRs of 1.4 or greater with HRT use after diagnosis for most cancer sites.

For peer review only

For peer review only - http://bmjopen.bmj.com/site/about/guidelines.xhtml

Table 4. Detectable hazard ratios for each type of cancer assuming 90% power to detect a difference and statistical significance at $p<0.05$

		HRT prior to diagnosis[1]		HRT post diagnosis analysis[2]	
Cancer type	Total patients	Total cancer specific deaths	Detectable hazard ratio	Cancer specific deaths after >6 months	Detectable hazard ratio
**Haematological**					
Leukaemia	8, 628	4, 323	1.20 (or 0.81)	2, 493	1.26 (or 0.75)
Multiple myeloma	5, 383	2, 839	1.24 (or 0.77)	2, 020	1.29 (or 0.72)
Non-Hodgkin's lymphoma	12, 584	5, 153	1.18 (or 0.83)	3, 271	1.23 (or 0.78)
**Gastro-intestinal**					
Oral	5, 855	2, 348	1.27 (or 0.74)	1, 761	1.31 (or 0.70)
Oesophageal	11, 464	8, 580	1.14 (or 0.86)	4, 409	1.19 (or 0.81)
Gastric	5, 580	4, 127	1.20 (or 0.80)	1, 998	1.29 (or 0.72)
Pancreatic	9, 941	8, 305	1.14 (or 0.86)	2, 679	1.25 (or 0.76)
Liver	3, 985	3, 176	1.23 (or 0.78)	1, 064	1.40 (or 0.62)
Colorectal	37, 930	17, 962	1.10 (or 0.91)	11, 398	1.12 (or 0.88)
**Gynaecological**					
Ovarian	16, 527	8, 942	1.13 (or 0.87)	5, 785	1.17 (or 0.83)
Endometrial	18, 857	5, 182	1.18 (or 0.83)	4, 550	1.19 (or 0.81)
Cervical	5, 594	1, 751	1.31 (or 0.70)	1, 263	1.37 (or 0.65)
**Urogenital**					
Kidney	7, 897	3, 530	1.22 (or 0.79)	2, 118	1.28 (or 0.73)
Bladder	10, 509	4, 429	1.19 (or 0.81)	3, 061	1.23 (or 0.77)
**Other**					
Brain	4, 760	3, 315	1.22 (or 0.78)	1, 362	1.35 (or 0.66)
Lung	40, 097	30, 391	1.07 (or 0.93)	13, 223	1.11 (or 0.89)
Thyroid	3, 878	553	1.57 (or 0.48)	324	1.75 (or 0.32)
Melanoma	16, 383	2, 553	1.26 (or 0.75)		1.27 (or 0.74)

HRT – Hormone Replacement Therapy

1. Uses total number of cancer specific deaths; 2. Uses number of deaths more than 6 months after diagnosis only

For peer review only - http://bmjopen.bmj.com/site/about/guidelines.xhtml

Ethics and dissemination

Ethical approval for this project was obtained from the QResearch scientific committee [Ref: OX24, project title “Use of hormone replacement therapy and survival from cancer”]. Ethical approval for the QResearch database is obtained annually from East Midlands - Derby Research Ethics Committee [Ref: 18/EM/0400].

The findings of this study will be submitted for peer-reviewed publication in an academic journal. In addition, the findings will be provided to Cancer Research UK for public dissemination, with the assistance of the Patient and Public Involvement (PPI) panel.

Patient and public involvement

The project will be supported throughout by a PPI panel, made up of women with experience of non-oestrogenic cancers. Members of the Northern Ireland Cancer Research Consumer Forum (NICRCF), a cancer patients’ group, provided feedback on the project proposal before submission to CRUK.

Another PPI panel of four women with a previous diagnosis of cancer was convened to inform the English component of this project (i.e. the one presented in this protocol). They agreed the study was important and potentially valuable for female cancer survivors who experience menopausal symptoms. The panel suggested that both cancer-specific mortality and subsequent cancer diagnoses would be of interest to women in this demographic considering whether to use HRT. Future discussions will seek feedback on the relevance and resonance of findings and guide us on research communications and dissemination.

For peer review only

References

1. Hamoda H, Panay N, Arya R, et al. The British Menopause Society & Women's Health Concern 2016 recommendations on hormone replacement therapy in menopausal women. *Post Reproductive Health* 2016;22(4):165-83. doi: 10.1177/2053369116680501
2. Goodman NF, Cobin RH, Ginzburg SB, et al. American Association of Clinical Endocrinologists Medical Guidelines for Clinical Practice for the diagnosis and treatment of menopause: executive summary of recommendations. *Endocr Pract* 2011;17(6):949-54. doi: 10.4158/ep.17.6.949 [published Online First: 2011/12/24]
3. Menon U, Burnell M, Sharma A, et al. Decline in use of hormone therapy among postmenopausal women in the United Kingdom. *Menopause* 2007;14(3 Pt 1):462-7. doi: 10.1097/01.gme.0000243569.70946.9d [published Online First: 2007/01/24]
4. Ameye L, Antoine C, Paesmans M, et al. Menopausal hormone therapy use in 17 European countries during the last decade. *Maturitas* 2014;79(3):287-91. doi: 10.1016/j.maturitas.2014.07.002
5. Million Women Study collaborators. Breast cancer and hormone-replacement therapy in the Million Women Study. *The Lancet* 2003;362(9382):419-27. doi: https://doi.org/10.1016/S0140-6736(03)14065-2
6. Cagnacci A, Venier M. The Controversial History of Hormone Replacement Therapy. *Medicina (Kaunas)* 2019;55(9) doi: 10.3390/medicina55090602 [published Online First: 2019/09/22]
7. Writing Group for the Women's Health Initiative I. Risks and Benefits of Estrogen Plus Progestin in Healthy Postmenopausal WomenPrincipal Results From the Women's Health Initiative Randomized Controlled Trial. *JAMA* 2002;288(3):321-33. doi: 10.1001/jama.288.3.321
8. Marjoribanks J, Farquhar C, Roberts H, et al. Long-term hormone therapy for perimenopausal and postmenopausal women. *Cochrane Database Syst Rev* 2017;1:CD004143. doi: 10.1002/14651858.CD004143.pub5 [published Online First: 2017/01/18]
9. Shen SS, Smith CL, Hsieh JT, et al. Expression of estrogen receptors-α and -β in bladder cancer cell lines and human bladder tumor tissue. *Cancer* 2006;106(12):2610-16. doi: 10.1002/cncr.21945
10. Wang X, Chen Q, Huang X, et al. Effects of 17β-estradiol and tamoxifen on gastric cancer cell proliferation and apoptosis and ER-α36 expression. *Oncol Lett* 2017;13(1):57-62. doi: 10.3892/ol.2016.5424 [published Online First: 2017/01/27]
11. Sukocheva OA, Wee C, Ansar A, et al. Effect of estrogen on growth and apoptosis in esophageal adenocarcinoma cells. *Dis Esophagus* 2013;26(6):628-35. doi: 10.1111/dote.12000 [published Online First: 2012/11/21]
12. Kanda N, Watanabe S. 17β-estradiol, Progesterone, and Dihydrotestosterone Suppress the Growth of Human Melanoma by Inhibiting Interleukin-8 Production. *Journal of Investigative Dermatology* 2001;117(2):274-83. doi: https://doi.org/10.1046/j.1523-1747.2001.01422.x
13. Hammoud Z, Tan B, Badve S, et al. Estrogen promotes tumor progression in a genetically defined mouse model of lung adenocarcinoma. *Endocr Relat Cancer* 2008;15(2):475-83. doi: 10.1677/erc-08-0002 [published Online First: 2008/05/30]
14. Ge H, Yan Y, Tian F, et al. Prognostic value of estrogen receptor α and estrogen receptor β in gastric cancer based on a meta-analysis and The Cancer Genome Atlas (TCGA) datasets. *Int J Surg* 2018;53:24-31. doi: 10.1016/j.ijsu.2018.03.027 [published Online First: 2018/03/21]
15. Cook MB, McGlynn KA, Devesa SS, et al. Sex disparities in cancer mortality and survival. *Cancer Epidemiol Biomarkers Prev* 2011;20(8):1629-37. doi: 10.1158/1055-9965.Epi-11-0246 [published Online First: 2011/07/14]
16. Radkiewicz C, Johansson ALV, Dickman PW, et al. Sex differences in cancer risk and survival: A Swedish cohort study. *Eur J Cancer* 2017;84:130-40. doi: 10.1016/j.ejca.2017.07.013 [published Online First: 2017/08/15]

For peer review only - http://bmjopen.bmj.com/site/about/guidelines.xhtml

17. Kim HW, Kim JH, Lim BJ, et al. Sex Disparity in Gastric Cancer: Female Sex is a Poor Prognostic Factor for Advanced Gastric Cancer. *Ann Surg Oncol* 2016;23(13):4344-51. doi: 10.1245/s10434-016-5448-0 [published Online First: 2016/11/03]
18. McGlynn KA, Hagberg K, Chen J, et al. Menopausal hormone therapy use and risk of primary liver cancer in the clinical practice research datalink. *International journal of cancer* 2016;138(9):2146-53. doi: 10.1002/ijc.29960 [published Online First: 2015/12/31]
19. Hassan MM, Botrus G, Abdel-Wahab R, et al. Estrogen Replacement Reduces Risk and Increases Survival Times of Women With Hepatocellular Carcinoma. *Clin Gastroenterol Hepatol* 2017;15(11):1791-99. doi: 10.1016/j.cgh.2017.05.036 [published Online First: 2017/06/01]
20. Simin J, Tamimi R, Lagergren J, et al. Menopausal hormone therapy and cancer risk: An overestimated risk? *European Journal of Cancer* 2017;84:60-68. doi: 10.1016/j.ejca.2017.07.012
21. Grodstein F, Newcomb PA, Stampfer MJ. Postmenopausal hormone therapy and the risk of colorectal cancer: a review and meta-analysis. *Am J Med* 1999;106(5):574-82. doi: 10.1016/s0002-9343(99)00063-7 [published Online First: 1999/05/21]
22. Mørch LS, Lidegaard Ø, Keiding N, et al. The influence of hormone therapies on colon and rectal cancer. *Eur J Epidemiol* 2016;31(5):481-9. doi: 10.1007/s10654-016-0116-z [published Online First: 2016/01/14]
23. Lope V, Fernández de Larrea N, Pérez-Gómez B, et al. Menstrual and Reproductive Factors and Risk of Gastric and Colorectal Cancer in Spain. *PLoS One* 2016;11(10):e0164620. doi: 10.1371/journal.pone.0164620 [published Online First: 2016/10/25]
24. Benson VS, Kirichek O, Beral V, et al. Menopausal Hormone Therapy and Risk of Central Nervous System Tumours: a UK-based Nested Case-Control Study. *International Journal of Epidemiology* 2015;44(suppl_1):i52-i52. doi: 10.1093/ije/dyv097.198
25. Jin C, Lang B. Hormone replacement therapy and lung cancer risk in women: a meta-analysis of cohort studies: Hormone replacement therapy and lung cancer risk. *Medicine (Baltimore)* 2019;98(51):e17532-e32. doi: 10.1097/MD.0000000000017532
26. Bae J-M, Kim EH. Hormonal Replacement Therapy and the Risk of Lung Cancer in Women: An Adaptive Meta-analysis of Cohort Studies. *J Prev Med Public Health* 2015;48(6):280-86. doi: 10.3961/jpmph.15.054 [published Online First: 2015/11/09]
27. Kane EV, Bernstein L, Bracci PM, et al. Postmenopausal hormone therapy and non-Hodgkin lymphoma: a pooled analysis of InterLymph case-control studies. *Ann Oncol* 2013;24(2):433-41. doi: 10.1093/annonc/mds340 [published Online First: 2012/09/13]
28. Costas L, Lujan-Barroso L, Benavente Y, et al. Reproductive Factors, Exogenous Hormone Use, and Risk of B-Cell Non-Hodgkin Lymphoma in a Cohort of Women From the European Prospective Investigation Into Cancer and Nutrition. *Am J Epidemiol* 2019;188(2):274-81. doi: 10.1093/aje/kwy259 [published Online First: 2018/11/28]
29. Morton LM, Wang SS, Richesson DA, et al. Reproductive factors, exogenous hormone use and risk of lymphoid neoplasms among women in the National Institutes of Health-AARP Diet and Health Study Cohort. *Int J Cancer* 2009;124(11):2737-43. doi: 10.1002/ijc.24248 [published Online First: 2009/03/03]
30. Daugherty SE, Lacey JJV, Pfeiffer RM, et al. Reproductive factors and menopausal hormone therapy and bladder cancer risk in the NIH-AARP Diet and Health Study. *International Journal of Cancer* 2013;133(2):462-72. doi: 10.1002/ijc.28022
31. Deli T, Orosz M, Jakab A. Hormone Replacement Therapy in Cancer Survivors – Review of the Literature. *Pathology & Oncology Research* 2020;26(1):63-78. doi: 10.1007/s12253-018-00569-x
32. Webber L, Anderson RA, Davies M, et al. HRT for women with premature ovarian insufficiency: a comprehensive review. *Hum Reprod Open* 2017;2017(2):hox007. doi: 10.1093/hropen/hox007 [published Online First: 2017/07/12]

For peer review only

For peer review only - http://bmjopen.bmj.com/site/about/guidelines.xhtml

33. Edey KA, Rundle S, Hickey M. Hormone replacement therapy for women previously treated for endometrial cancer. *Cochrane Database of Systematic Reviews* 2018(5) doi: 10.1002/14651858.CD008830.pub3
34. Ji J, Sundquist J, Sundquist K. Use of hormone replacement therapy improves the prognosis in patients with colorectal cancer: A population-based study in Sweden. *Int J Cancer* 2018;142(10):2003-10. doi: 10.1002/ijc.31228 [published Online First: 2017/12/23]
35. Chan JA, Meyerhardt JA, Chan AT, et al. Hormone replacement therapy and survival after colorectal cancer diagnosis. *J Clin Oncol* 2006;24(36):5680-6. doi: 10.1200/jco.2006.08.0580 [published Online First: 2006/12/21]
36. Jang YC, Huang HL, Leung CY. Association of hormone replacement therapy with mortality in colorectal cancer survivor: a systematic review and meta-analysis. *BMC Cancer* 2019;19(1):1199. doi: 10.1186/s12885-019-6428-0 [published Online First: 2019/12/11]
37. MacKie RM, Bray CA. Hormone replacement therapy after surgery for stage 1 or 2 cutaneous melanoma. *Br J Cancer* 2004;90(4):770-2. doi: 10.1038/sj.bjc.6601595 [published Online First: 2004/02/19]
38. Krul IM, Opstal-van Winden AWJ, Aleman BMP, et al. Breast Cancer Risk After Radiation Therapy for Hodgkin Lymphoma: Influence of Gonadal Hormone Exposure. *Int J Radiat Oncol Biol Phys* 2017;99(4):843-53. doi: 10.1016/j.ijrobp.2017.07.016 [published Online First: 2017/09/11]
39. Yang X, Wang C, He X, et al. Hormone therapy for premature ovarian insufficiency patients with malignant hematologic diseases. *Climacteric* 2017;20(3):268-73. doi: 10.1080/13697137.2017.1309382 [published Online First: 2017/04/13]
40. Ganti AK. Another nail in the coffin for hormone-replacement therapy? *The Lancet* 2009;374(9697):1217-18. doi: https://doi.org/10.1016/S0140-6736(09)61571-3
41. Biglia N, Gadducci A, Ponzone R, et al. Hormone replacement therapy in cancer survivors. *Maturitas* 2004;48(4):333-46. doi: https://doi.org/10.1016/j.maturitas.2003.09.031
42. Cobin RH, Goodman NF, Committee ARES. American Association of Clinical Endocrinologists and American College of Endocrinology position statement on menopause–2017 update. *Endocrine practice* 2017;23(7):869-80.
43. National Institute for Health and Care Excellence. Menopause: diagnosis and management2019.
44. Welton AJ, Vickers MR, Kim J, et al. Health related quality of life after combined hormone replacement therapy: randomised controlled trial. *BMJ* 2008;337:a1190. doi: 10.1136/bmj.a1190
45. Oliver-Williams C, Glisic M, Shahzad S, et al. The route of administration, timing, duration and dose of postmenopausal hormone therapy and cardiovascular outcomes in women: a systematic review. *Hum Reprod Update* 2019;25(2):257-71. doi: 10.1093/humupd/dmy039 [published Online First: 2018/12/07]
46. Kontopantelis E, Stevens RJ, Helms PJ, et al. Spatial distribution of clinical computer systems in primary care in England in 2016 and implications for primary care electronic medical record databases: a cross-sectional population study. *BMJ Open* 2018;8(2):e020738. doi: 10.1136/bmjopen-2017-020738
47. Hippisley-Cox J, Stables D Fau - Pringle M, Pringle M. QRESEARCH: a new general practice database for research. (1476-0320 (Print))
48. British Medical Association Joint Formulary Committee Pharmaceutical Society (Great Britain). British national formulary: Pharmaceutical Pr 2003.
49. Vinogradova Y, Coupland C, Hippisley-Cox J. Use of hormone replacement therapy and risk of venous thromboembolism: nested case-control studies using the QResearch and CPRD databases. *Bmj* 2019 doi: 10.1136/bmj.k4810

For peer review only - http://bmjopen.bmj.com/site/about/guidelines.xhtml

50. Arık A, Dodd E, Streftaris G. Cancer morbidity trends and regional differences in England—A Bayesian analysis. *PLOS ONE* 2020;15(5):e0232844. doi: 10.1371/journal.pone.0232844
51. Public Health England. Linking treatment tables - chemotherapy, tumour resections and radiotherapy. 4.4 ed: Cancer Research UK - Public Health England, 2018:58.
52. Suissa S, Azoulay L. Metformin and the risk of cancer: time-related biases in observational studies. *Diabetes Care* 2012;35(12):2665-73. doi: 10.2337/dc12-0788 [published Online First: 2012/11/23]
53. White IR, Royston P, Wood AM. Multiple imputation using chained equations: Issues and guidance for practice. *Stat Med* 2011;30(4):377-99. doi: 10.1002/sim.4067 [published Online First: 2011/01/13]
54. Rubin DB. Multiple imputation for nonresponse in surveys: John Wiley & Sons 2004.
55. Registries UKaIAoC. UKIACR: Performance Indicators 2018 Report, 2018.
56. Ji J, Sundquist J, Sundquist K. Use of hormone replacement therapy improves the prognosis in patients with colorectal cancer: A population-based study in Sweden. *International Journal of Cancer* 2018;142(10):2003-10. doi: 10.1002/ijc.31228
57. Hicks B, Kristensen KB, Pedersen SA, et al. Hormone replacement therapy and the risk of melanoma in post-menopausal women. *Human Reproduction* 2019;34(12):2418-29.

For peer review only

For peer review only - http://bmjopen.bmj.com/site/about/guidelines.xhtml

Author's contributions

All other authors made significant contributions to the compilation of the manuscript.

Sources of funding

This work was supported by Cancer Research UK [grant number: C37316/A29656]

The study sponsor has no role in: study design; collection, management, analysis or interpretation of data; writing of the report; or the decision to submit the report for publication.

JHC has received grants from the National Institute for Health Research Biomedical Research Centre, Oxford, John Fell Oxford University Press Research Fund, Cancer Research UK (grant number C5255/A18085) through the Cancer Research UK Oxford Centre, and the Oxford Wellcome Institutional Strategic Support Fund (204826/Z/16/Z) during the conduct of the study.

AC is supported by a Clinical Research Training Fellowship from Cancer Research UK (DCS-CRUK-CRTF20-AC).

Competing interests statement

JHC is an unpaid director of the Qresearch database (partnership between the University of Oxford and EMIS, commercial supplier of GP computer systems). She is shareholder and former director of ClinRisk Ltd outside the submitted work.

PST reports consulting with AstraZeneca and Duke-NUS outside the submitted work.

The remaining authors declare no competing interests.

Word count

6,748

For peer review only

STROBE 2007 (v4) Statement—Checklist of items that should be included in reports of *cohort studies*

Section/Topic	Item #	Recommendation	Reported on page #
**Title and abstract**	1	(*a*) Indicate the study's design with a commonly used term in the title or the abstract	1
		(*b*) Provide in the abstract an informative and balanced summary of what was done and what was found	2
**Introduction**			
Background/rationale	2	Explain the scientific background and rationale for the investigation being reported	4-6
Objectives	3	State specific objectives, including any prespecified hypotheses	7
**Methods**			
Study design	4	Present key elements of study design early in the paper	7
Setting	5	Describe the setting, locations, and relevant dates, including periods of recruitment, exposure, follow-up, and data collection	7
Participants	6	(*a*) Give the eligibility criteria, and the sources and methods of selection of participants. Describe methods of follow-up	7
		(*b*) For matched studies, give matching criteria and number of exposed and unexposed	n/a
Variables	7	Clearly define all outcomes, exposures, predictors, potential confounders, and effect modifiers. Give diagnostic criteria, if applicable	8-13
Data sources/ measurement	8*	For each variable of interest, give sources of data and details of methods of assessment (measurement). Describe comparability of assessment methods if there is more than one group	8-13
Bias	9	Describe any efforts to address potential sources of bias	16, 17
Study size	10	Explain how the study size was arrived at	18, 19
Quantitative variables	11	Explain how quantitative variables were handled in the analyses. If applicable, describe which groupings were chosen and why	13- 15
Statistical methods	12	(*a*) Describe all statistical methods, including those used to control for confounding	13- 15
		(*b*) Describe any methods used to examine subgroups and interactions	14- 16
		(*c*) Explain how missing data were addressed	17
		(*d*) If applicable, explain how loss to follow-up was addressed	n/a
		(*e*) Describe any sensitivity analyses	16

For peer review only

**Results – CHECKLIST NOT COMPLETED BEYOND THIS POINT AS THE CURRENT SUBMISSION IS A PROTOCOL ONLY**			
Participants	13*	(a) Report numbers of individuals at each stage of study—eg numbers potentially eligible, examined for eligibility, confirmed eligible, included in the study, completing follow-up, and analysed	n/a
		(b) Give reasons for non-participation at each stage	n/a
		(c) Consider use of a flow diagram	n/a
Descriptive data	14*	(a) Give characteristics of study participants (eg demographic, clinical, social) and information on exposures and potential confounders	n/a
		(b) Indicate number of participants with missing data for each variable of interest	n/a
		(c) Summarise follow-up time (eg, average and total amount)	n/a
Outcome data	15*	Report numbers of outcome events or summary measures over time	n/a
Main results	16	(*a*) Give unadjusted estimates and, if applicable, confounder-adjusted estimates and their precision (eg, 95% confidence interval). Make clear which confounders were adjusted for and why they were included	n/a
		(*b*) Report category boundaries when continuous variables were categorized	n/a
		(*c*) If relevant, consider translating estimates of relative risk into absolute risk for a meaningful time period	n/a
Other analyses	17	Report other analyses done—eg analyses of subgroups and interactions, and sensitivity analyses	n/a
**Discussion**			
Key results	18	Summarise key results with reference to study objectives	n/a
**Limitations**			
Interpretation	20	Give a cautious overall interpretation of results considering objectives, limitations, multiplicity of analyses, results from similar studies, and other relevant evidence	n/a
Generalisability	21	Discuss the generalisability (external validity) of the study results	n/a
**Other information**			
Funding	22	Give the source of funding and the role of the funders for the present study and, if applicable, for the original study on which the present article is based	n/a

*Give information separately for cases and controls in case-control studies and, if applicable, for exposed and unexposed groups in cohort and cross-sectional studies.

Note: An Explanation and Elaboration article discusses each checklist item and gives methodological background and published examples of transparent reporting. The STROBE checklist is best used in conjunction with this article (freely available on the Web sites of PLoS Medicine at http://www.plosmedicine.org/, Annals of Internal Medicine at http://www.annals.org/, and Epidemiology at http://www.epidem.com/). Information on the STROBE Initiative is available at www.strobe-statement.org.

For peer review only

Hormone Replacement Therapy and Cancer Survival: A Longitudinal Cohort Study - protocol paper

Journal:	*BMJ Open*
Manuscript ID	bmjopen-2020-046701.R1
Article Type:	Protocol
Date Submitted by the Author:	19-Apr-2021
Complete List of Authors:	Ranger, Tom; Oxford University, Nuffield Department of Primary Care Health Sciences<br>Burchardt, Judith; Oxford University, Nuffield Department of Primary Care Health Sciences<br>Clift, Ashley; University of Oxford, Nuffield Department of Primary Care Health Sciences<br>Mei, Xue; Oxford University, Nuffield Department of Primary Care Health Sciences<br>Coupland, Carol; University of Nottingham, Division of Primary Care<br>Tan, Pui San; University of Oxford, Nuffield Department of Primary Care Health Sciences<br>Dixon, Sharon; University of Oxford, Nuffield Department of Primary Care Health Sciences<br>Cardwell, Christopher; Queen's University Belfast, School of Medicine, Dentistry and Biomedical Sciences<br>Hippisley-Cox, Julia; University of Oxford, Nuffield Department of Primary Care Sciences
**Primary Subject Heading**:	Public health
Secondary Subject Heading:	Epidemiology, Evidence based practice, General practice / Family practice, Public health, Oncology
Keywords:	EPIDEMIOLOGY, ONCOLOGY, Sex steroids & HRT < DIABETES & ENDOCRINOLOGY

SCHOLARONE™
Manuscripts

BMJ

I, the Submitting Author has the right to grant and does grant on behalf of all authors of the Work (as defined in the below author licence), an exclusive licence and/or a non-exclusive licence for contributions from authors who are: i) UK Crown employees; ii) where BMJ has agreed a CC-BY licence shall apply, and/or iii) in accordance with the terms applicable for US Federal Government officers or employees acting as part of their official duties; on a worldwide, perpetual, irrevocable, royalty-free basis to BMJ Publishing Group Ltd ("BMJ") its licensees and where the relevant Journal is co-owned by BMJ to the co-owners of the Journal, to publish the Work in this journal and any other BMJ products and to exploit all rights, as set out in our licence.

The Submitting Author accepts and understands that any supply made under these terms is made by BMJ to the Submitting Author unless you are acting as an employee on behalf of your employer or a postgraduate student of an affiliated institution which is paying any applicable article publishing charge ("APC") for Open Access articles. Where the Submitting Author wishes to make the Work available on an Open Access basis (and intends to pay the relevant APC), the terms of reuse of such Open Access shall be governed by a Creative Commons licence – details of these licences and which Creative Commons licence will apply to this Work are set out in our licence referred to above.

Other than as permitted in any relevant BMJ Author's Self Archiving Policies, I confirm this Work has not been accepted for publication elsewhere, is not being considered for publication elsewhere and does not duplicate material already published. I confirm all authors consent to publication of this Work and authorise the granting of this licence.

For peer review only

Hormone Replacement Therapy and Cancer Survival:

A Longitudinal Cohort Study - protocol paper

Tom A. Ranger[1], Judith Burchardt[1], Ashley K. Clift[1,2], Winnie Mei[1], Carol Coupland[3], Pui San Tan[1], Sharon Dixon[1], Christopher R. Cardwell[4], Julia Hippisley-Cox[1]

1. Primary Care Epidemiology, Nuffield Department of Primary Care Health Sciences, Medical Sciences Division, University of Oxford, Oxford, UK
2. Cancer Research UK Oxford Centre, Department of Oncology, Old Road Campus Research Building, Roosevelt Drive, Oxford, UK
3. Division of Primary Care, University of Nottingham, Nottingham, UK
4. Cancer Epidemiology and Health Services Research Group (CRC, MMC), Centre for Public Health, Queen's University Belfast, Royal Victoria Hospital Site, Belfast, UK

For peer review only

Running head: HRT and cancer survival

Date and version: March 2021, version 2

Corresponding author:

Dr. Tom Ranger, PhD

Email: tom.ranger@phc.ox.ac.uk
Nuffield Department of Primary Care Health Sciences,
Radcliffe Primary Care Building,
University of Oxford,
Oxford, UK

For peer review only - http://bmjopen.bmj.com/site/about/guidelines.xhtml

Abstract

Introduction

Hormone replacement therapy (HRT) can help women experiencing menopausal symptoms, but usage has declined due to uncertainty around risks of cancer and some cardio-vascular diseases (CVD). Moreover, improved cancer survival rates mean that more women who survive cancer go on to experience menopausal symptoms. Understanding these relationships is important so that women and their clinicians can make informed decisions around the risks and benefits of HRT.

This study's primary aim is to determine the association between HRT use after cancer diagnosis and the risk of cancer-specific mortality. The secondary aims are to investigate the risks of HRT on subsequent cancer, all-cause mortality and CVD.

Methods and analysis

We will conduct a population-based longitudinal cohort study of 18 to 79-year-old women diagnosed with cancer between 1998 and 2020, using the QResearch database. The main exposure is HRT use, categorised based on compound, dose and route of administration, and modeled as a time-varying covariate. Analysis of HRT use pre- and post-cancer diagnosis will be conducted separately.

The primary outcome is cancer-specific mortality, which will be stratified by cancer site. Secondary outcomes include subsequent cancer diagnosis, CVD (including venous thrombo-embolism) and all-cause mortality. Adjustment will be made for key confounders such as age, BMI, ethnicity, deprivation index, comorbidities, and cancer grade, stage and treatment. Statistical analysis will include descriptive statistics and Cox proportional hazards models to calculate hazard ratios and 95% confidence intervals.

Ethics and dissemination

Ethical approval for this project was obtained from the QResearch Scientific Committee [Ref: OX24, project title "Use of hormone replacement therapy and survival from cancer"]. This project has been, and will continue to be, supported by patient and public involvement panels. We intend to the submit the findings for peer-reviewed publication in an academic journal and disseminate them to the public through Cancer Research UK.

For peer review only - http://bmjopen.bmj.com/site/about/guidelines.xhtml

Strengths and limitations of this study

- This is an open cohort study comprising a nationally representative sample of English women.
- The cohort consists of GP clinic data linked to hospital records, the English national cancer registry and English national death registry.
- This study has access to detailed information on HRT prescriptions, allowing analysis with consideration of the specific compound, dose, route of administration and duration of exposure.
- This study is limited by high rates of missing data for cancer grade and stage, although completeness has improved in recent years, this will be accounted for using appropriate multiple imputation techniques.

For peer review only

For peer review only - http://bmjopen.bmj.com/site/about/guidelines.xhtml

Background

Prevalence of HRT use

Hormone replacement therapy (HRT) involves clinical provision of the female sex hormone oestrogen either on its own or in combination with another hormone, progestogen. It is an effective and important treatment for symptoms of the menopause, including vasomotor symptoms (hot flushes and night sweats), urogenital atrophy[1] and postmenopausal osteoporosis[2]. Yet between 2002 and 2005, HRT usage in the UK dropped from approximately 29% of postmenopausal women to approximately 11%[3], and this pattern was consistent across Europe[4]. The drop in usage has been attributed to the widely reported findings of the Million Women's Study[5] and the early cessation of the Women's Health Initiative trial[3 4 6], which concluded that an observed increase in risk of cardiovascular events and breast cancer in women taking combined oestrogen and progestogen HRT outweighed the benefits of reduced rates of colorectal cancer and osteoporotic fractures[7]. Evidence from a recent Cochrane systematic review and meta-analysis though, suggests that HRT remains an important treatment option for women with severe menopausal symptoms who do not have other risk factors, such as oestrogen sensitive cancers[8]. Given the demonstrated risks and benefits of HRT, there is a need to clarify which women can use it safely, to treat menopausal symptoms without unduly increasing their risk of adverse outcomes.

HRT in women with cancer

The role of oestrogen in cancer is complex, with evidence that it accelerates cancer progression in some sites but slows it in others. For instance, pre-clinical studies have shown that oestrogen stimulates growth in bladder[9] and gastric cancer cell lines[10] but, in contrast, similar studies found that it inhibits cell growth and/or induces apoptosis in oesophageal adenocarcinoma[11] and malignant melanoma cell lines[12]. What's more, in a mouse model oestrogen increased the number and volume of lung adenocarcinomas[13], and molecular pathological studies have shown oestrogen receptor expression is associated with metastasis and poorer survival in gastric cancer[14]. Epidemiological studies also report contrasting findings depending the cancer of interest, with some showing markedly lower incidence and slightly better survival for women compared to men for many cancers[15 16], whereas another study demonstrated survival after gastric cancer is worse in young women (<45 years old) than in young men, but similar in older women and men[17]. The complex relationship between oestrogen and cancer raises questions as to how HRT can be safely provided for women experiencing menopausal symptoms.

For peer review only - http://bmjopen.bmj.com/site/about/guidelines.xhtml

Similar to investigations of oestrogen's role in various cancers, findings from studies of HRT and cancer risk are mixed. For example, HRT use is associated with a reduced risk of liver[18-20], oesophageal[20] and colorectal cancers[20-23] but an increased risk of glioma and meningioma with use of oestrogen only HRT[24]. In some cases evidence is conflicting for individual cancers, for example, a recent meta-analysis of lung cancer found a reduced risk in women taking HRT[25] but an earlier meta-analysis found no effect[26]; and in non-Hodgkin lymphoma, for which a pooled analysis of case-control studies showed decreased incidence in women using HRT[27], but two cohort studies found no effect[28 29]. Furthermore, a cohort study found bladder cancer to be less common in women using combined, but not oestrogen-only HRT[30], suggesting that the specific HRT prescription affects its association with cancer.

There are also important questions relating to the risk of HRT use by women who have survived or are living with cancer. This is increasingly important for several reasons: first, improved survival rates from cancer mean female survivors are increasingly likely to experience menopause[31]; second, in young women with cancer, the associated treatment may cause primary ovarian insufficiency (POI)[31 32], inducing menopause and thus potentially indicating HRT in this demographic; and third, the induction of 'surgical' menopause by hysterectomy and oophorectomy could be an indication for HRT[33]. While a number of studies have found protective effects of HRT after colorectal cancer[34-36], epidemiological evidence at other cancer sites is sparse. For instance, HRT use after cancer diagnosis was associated with reduced mortality in 205 melanoma patients[37], but this was based upon only one cancer-specific death in HRT users. Similarly, HRT before diagnosis was associated with reduced mortality in 234 patients with primary liver cancer, but HRT after diagnosis was not investigated[19]. A case control study of Hodgkin's lymphoma patients with and without subsequent breast cancer did not find that risk was increased by HRT[38]. Finally, in a study of 130 leukaemia patients no excess recurrences were observed in HRT users[39]. Further investigation into the potential association between HRT and cancer survival is warranted because existing evidence comes from small studies with limited power, many of which did not investigate HRT after diagnosis, and many cancer sites have not been investigated at all.

Despite the lack of high quality epidemiological evidence there have been suggestions that HRT should be used cautiously, or not at all, in patients with lung[31 40], bladder[31 41], gastric[31 41], and brain cancer[31 41]. These recommendations may be concerning for patients, and for clinicians there is limited information on providing HRT in women with cancers other than breast cancer in clinical guidelines[42 43]. This can lead to avoidance of HRT in line with the 'precautionary principle', but such an approach has been severely criticized because of the clear benefits of HRT in reducing menopausal symptoms and increasing health

For peer review only - http://bmjopen.bmj.com/site/about/guidelines.xhtml

related quality of life[31 44]. There is a need to clarify the relationship between HRT and non-oestrogen dependent cancers so that women of all ages who survive cancer can make informed choices with their treating clinicians as to the risks and benefits of HRT.

Risk of cardiovascular disease and venous thrombo-embolism associated with HRT

In addition to cancer, there are concerns that HRT may lead to cardiovascular complications. A recent Cochrane systematic review with meta-analysis of 22 largely high quality studies found moderate evidence that combined, continuous HRT is associated with an increased risk of myocardial infarction (MI), stroke and venous thromboembolism (VTE), and that oestrogen only, continuous HRT is associated with stroke and VTE but not MI[8]. The authors concluded that while the absolute risk of these outcomes remained low, the clear association meant that the increased relative risk is a relevant consideration for all women and may contraindicate HRT in those at increased risk of cardiovascular disease (CVD). A more recent systematic review of 33 observational studies, however, had contradictory findings that HRT may protect against CVD, and that the duration, dose, timing and means of HRT administration affected the relationship[45]. Importantly, the authors noted inconsistent results in the included studies and a wide range in their overall scientific quality. The higher quality of studies included in the Cochrane review lend greater weight to its findings, but the more recent systematic review indicates that further research into the specific type and administration of HRT in high quality studies is needed.

Summary

There are legitimate concerns about the safety of prescribing HRT for women with cancer and those at elevated risk of VTE or CVD. The role of HRT in improving the quality of life for women during menopause, however, means that understanding how and for whom it can be safely prescribed is essential. Furthermore, the increasing number of women who survive cancer and go on to experience menopausal symptoms suggests that investigation is warranted into this demographic specifically.

Aims and objectives

For peer review only

For peer review only - http://bmjopen.bmj.com/site/about/guidelines.xhtml

Our primary aim is to determine the association between HRT use and the risk of cancer-specific mortality in women with a range of common cancers. Our secondary aim is to quantify the risks of HRT associated with subsequent cancer, all-cause mortality, CVD and VTE in women with cancer. Our study will provide cancer patients and clinicians with the high quality evidence of the safety of HRT in cancer patients, allowing them to make informed decisions, and will provide important mechanistic insights into the role of oestrogen in cancer progression.

Methods and analysis

Study design and data source

A population-based cohort study will be conducted using the QResearch linked database. QResearch is a large, validated database of anonymized electronic health records and is representative of the English general population[46]. It compiles patient data at an individual level from GP practices through the Egton Medical Information Systems (EMIS) electronic health records and has linkages to the NHS Digital Hospital Episode Statistics (HES) platform, the English national cancer registry and the English national death register, and has been described in detail previously[47].

Study sample

We will identify an open cohort of women aged 18 to 79 years who have been registered at a GP clinic contributing data to QResearch for at least 1 year prior to diagnosis of one of the included types of cancer (Table 1). Women with cancer will be identified from the English national cancer registry and this will be supplemented with GP clinic records and HES data. The study period is between 1998 and the most recent available data, with censoring at either the date of death or the end of the study period in the primary analysis, and at development of subsequent cancer, CVD or VTE in the relevant secondary analyses. Exclusion criteria are: women with a previous diagnosis of invasive cancer (other than those listed) or who have less than one year of follow-up available in the database. Breast cancer will not be included since HRT is contraindicated in breast cancer and oestrogen dependent tumours[2 42 48].

Table 1. Included cancer sites of primary tumours by classification

For peer review only - http://bmjopen.bmj.com/site/about/guidelines.xhtml

Category	Specific Tumour Site
Haematological	Leukaemia, multiple myeloma, non-Hodgkin's lymphoma
Gastro-intestinal	Oral, oesophageal, gastric, liver, pancreatic, colorectal
Gynaecological	Cervical, ovarian, endometrial
Urogenital	Renal, bladder
Other cancer sites	Malignant melanoma, lung, thyroid, brain

For peer review only

Exposure

We will extract data on HRT prescriptions from the QResearch database. Treatment will be categorized based on compound (type of oestrogen and type of progestogen where applicable), route of administration (oral, transdermal, vaginal, injection or implant), dose (≤0.625 mg for oral conjugated equine oestrogen, ≤1 mg for oral estradiol, ≤50 µg for transdermal estradiol[49]), timing (i.e. relative to cancer diagnosis) and duration. Daily defined doses (DDDs) will be extrapolated from prescription information where possible, however, it may not be possible in some cases, such as administration by transdermal patch or gel.

Outcomes

Outcomes related to cancer mortality

The primary outcome is cancer specific mortality where cancer is recorded as the primary or underlying cause of death. This will be ascertained using the primary or underlying cause of death as recorded on the linked mortality data from the Office of National Statistics death register. Our secondary outcomes will be incident cases of cancer as recorded on either the GP, hospital, cancer registry or linked mortality record, and all-cause mortality.

Outcomes related to CVD and VTE

A CVD event will be defined as the earliest record of CVD where it is the primary reason for admission on the GP or HES databases, or where it is recorded as primary cause of death on the mortality record. A CVD event will include coronary heart disease or stroke.

For peer review only - http://bmjopen.bmj.com/site/about/guidelines.xhtml

As for CVD, a VTE event will be defined as the earliest record of VTE on any of the three linked data sources (GP, hospital episode statistics or mortality record). VTE will include deep vein thrombosis or pulmonary embolism.

Confounding variables

Key demographic factors, potentially confounding factors and data relating to the secondary outcome (CVD) are listed below and will be obtained from GP records. Categories of confounders that may apply to both outcomes but with different specific inclusions (e.g. pharmacotherapy) are listed in Table 2, with specific inclusions shown separately for each outcome.

Demographic and lifestyle factors

- Year of birth
- Age at cancer diagnosis
- Townsend deprivation score
- Most recent Body Mass Index (BMI) prior to outcome diagnosis
- Family history of cancer
- Ethnicity (White, Indian, Bangladeshi, Pakistani, Chinese, other Asian, Caribbean, Black African, other)
- Most recent smoking status (never, former, light, moderate, heavy) prior to outcome diagnosis
- Most recent alcohol consumption status (non-drinker, trivial, light, moderate, heavy, very heavy) prior to outcome diagnosis
- Geographical region (East of England, East Midlands, West Midlands, London, North East, North West, South Central, South East, South West, Yorkshire and the Humber)[50]

For peer review only - http://bmjopen.bmj.com/site/about/guidelines.xhtml

Table 2. Categories of confounders with specific inclusions for each outcome shown separately

	**Cancer related analyses**	**CVD and VTE related analyses**
Charlson Comorbidity Index (CCI) conditions	Cardiovascular diseases<br>• Coronary heart disease (myocardial infarction or angina)<br>• Congestive cardiac failure<br>• Peripheral vascular disease<br>• Stroke/ transient ischaemic attack<br>Gastro-intestinal conditions<br>• Peptic ulcer disease<br>• Liver disease<br>• Inflammatory bowel disease<br>Other CCI conditions<br>• Dementia<br>• Chronic obstructive pulmonary disease<br>• Connective tissue disease<br>• Hemiplegia<br>• Moderate or severe chronic kidney disease<br>• Diabetes mellitus (type 1 or type 2)<br>• HIV/ AIDS	Gastro-intestinal conditions<br>• Peptic ulcer disease<br>• Liver disease<br>• Inflammatory bowel disease<br>Other CCI conditions<br>• Dementia<br>• Chronic obstructive pulmonary disease<br>• Connective tissue disease<br>• Hemiplegia<br>• Moderate or severe chronic kidney disease<br>• Diabetes mellitus (type 1 or type 2)<br>• HIV/ AIDS
Other comorbidities	• Severe mental illness	• Severe mental illness

For peer review only - http://bmjopen.bmj.com/site/about/guidelines.xhtml

For peer review only

	• Depression<br>• Venous thromboembolism<br>• VBI syndrome<br>• Anaemia	• Depression<br>• Anaemia
Pharmacotherapy (other than HRT and chemotherapy)	• Aspirin<br>• Statins<br>• Losartan<br>• Metformin	• Anticoagulant drugs - warfarin, oral and parenteral anticoagulants<br>• Antihypertensive drugs – ACE-inhibitors, beta-blockers, calcium channel blockers, diuretics<br>• Antiplatelet drugs – aspirin, clopidogrel, dipyridamole, prasugrel, ticagrelor, ticlopidine<br>• Mental health drugs<br>○ Antidepressants - tricyclic antidepressants, compound antidepressants, mono-amine oxidase inhibitors<br>○ Antipsychotic drugs<br>• Analgesic drugs<br>○ Non-steroidal anti-inflammatories<br>○ Opioids<br>• Drugs of cancer treatment - tamoxifen, aromatase inhibitors, thalidomide, lenalidomide, bevacizumab

For peer review only - http://bmjopen.bmj.com/site/about/guidelines.xhtml

		• Immunosuppressant drugs<br>• Antidiabetes drugs - insulin, oral hypoglycaemics<br>• Prostaglandin analogues<br>• Other hormone therapies<br>○ Oral contraceptive drugs - dianette<br>○ Topical hormone drugs<br>○ Megestrol acetate

For peer review only - http://bmjopen.bmj.com/site/about/guidelines.xhtml

Tumour specific factors

Histology

- Stage: 1-4 as per Tumour Nodes Metastases classification, or FIGO for gynaecological cancers
- Grade: well, moderately, poorly or not differentiated

Cancer treatment within 6 months of diagnosis[51]

- Radiotherapy
- Chemotherapy
- Hormonal therapy for cancer as recorded on the cancer registry
- Cancer related surgery
    - Lung – resection, e.g. pneumonectomy or lobectomy
    - Colorectal – hemicolectomy, colectomy, endoscopic resection
    - Pancreatic – pancreaticoduodenectomy, distal pancreatectomy
    - Gynaecological – hysterectomy with or without bilateral salpingo-oophorectomy, pelvic exenterations, cone biopsy or LLTEZ
    - Gastro-oesophageal – oesophagectomy, gastrectomy
    - Oral – lip resection, excision of oral lesion, glossectomy, maxillectomy, mandibulectomy, laryngectomy
    - Thyroid cancers – thyroidectomy with or without neck dissection
    - Urogenital – nephrectomy, nephroureterectomy, cystectomy
    - Liver cancers – segmental resection, lobectomy, transplantation
    - Brain cancers – craniectomy with resection

For peer review only

Other surgery (i.e. not cancer related)

- Hysterectomy with or without oophorectomy

Statistical analysis

Analysis of HRT use after cancer diagnosis

Patients will be followed from 6 months after cancer diagnosis to cancer-specific mortality (censored on death from other causes or end of follow-up). A delay such as this is recommended to exclude deaths

For peer review only - http://bmjopen.bmj.com/site/about/guidelines.xhtml

related to severe pathology to which HRT use is unlikely to have contributed. The length of the delay will be varied for certain cancers (such as melanoma, non-Hodgkin's lymphoma, bladder, colorectal and thyroid cancer) as they have better prognosis.

A dose-response analysis will be conducted based upon DDD's, and number and duration of prescriptions. To avoid immortal time bias, HRT use will be modelled as a time varying covariate, i.e. patients will only be considered users after a lag of 6 months from their first HRT prescription. Therefore, individuals will be considered users of 0 to 1 year from 6 months after their first prescription to 6 months after their 12th prescription (or 365th DDD) and >1 year users after this time (Table 3A). Other elements of HRT prescription, namely dose, means of administration and whether administration is continuous or cyclical will be investigated through stratification of the sample.

Time-dependent Cox regression models will be used to calculate Hazard Ratios (HRs), and 95% Confidence Intervals (CIs), for HRT use after cancer diagnosis adjusting for potential confounders including: age at diagnosis, BMI, year of diagnosis, alcohol and smoking status, ethnicity, deprivation index, stage, grade, cancer treatment, comorbidities, routine medications and prior hysterectomy/ oophorectomy.

Analysis of HRT use before cancer diagnosis

Patients will be followed from diagnosis to cancer-specific mortality. HRT use will be identified in the 18 months prior to diagnosis, excluding the 6 months immediately prior to diagnosis (Table 3B). Exclusion of HRT use in this period is intended to avoid bias arising from potentially short-term prescriptions issued in the lead up to cancer diagnosis that are unlikely to affect the outcome and may result in misclassification[52]. Cox regression models will be used to calculate HRs and 95% CIs for cancer-specific death in HRT users compared with non-users. Adjusted Cox regression models will include: age at diagnosis, BMI, year of diagnosis, alcohol and smoking status, ethnicity, deprivation index, comorbidities, prior hysterectomy/oophorectomy and routine medication use before diagnosis. Stage, grade and cancer treatments will initially be excluded from the models investigating HRT before diagnosis, as they could lie on the causal pathway. Subsequent analysis, however, will explore their potential role as confounders or effect mediators. Similar analyses will be conducted for secondary outcomes.

For peer review only - http://bmjopen.bmj.com/site/about/guidelines.xhtml

Confounding factors for specific cancer sites

Analyses will be stratified by cancer site, and some sites will be adjusted for additional covariates that have been shown to be associated with outcome at those sites. For instance, analysis of lung cancer will additionally contain histology (small-cell/non-small-cell) and beta-blocker use; colorectal will additionally contain site (colon, rectum or rectosigmoid colon), family history of colorectal cancer and inflammatory bowel disease; and oesophageal will contain histology (adenocarcinoma and squamous cell).

Analysis of CVD

In the secondary analysis of cardiovascular disease, a start/ stop time-varying covariate analysis will be conducted to investigate current HRT use, in which patients will become HRT users on the date of each HRT prescription, and remain HRT users until the end of their current prescription, at which point they will become past HRT users (Table 3C) unless they initiate another HRT treatment within 30 days.

Cox regression models will be used to calculate HRs and 95%CIs for CVD events in HRT users and non-users. Adjustment will be made for: age at diagnosis, BMI, ethnicity, alcohol and smoking status, year of diagnosis, comorbidities and medications.

Table 3A-C. Categorisation of HRT users for each proposed analysis

**Label**	**Definition**
**3A.** HRT use *after* cancer diagnosis	
Non-user	No HRT prescription, or <6 months from first HRT prescription
0-1 year user	6 months after 1st prescription until 6 months after 12th prescription or 6 months after 365th DDD
>1 year user	Prescriptions beyond above time points
**3B.** HRT use *before* cancer diagnosis	
Non-user	No HRT prescription in the period from 18 months to 6 months before cancer diagnosis
User	HRT prescription in the period from 18 months to 6 months before cancer diagnosis
**3C.** HRT use and CVD	
Non-user	A woman without an HRT prescription
Current user	A woman with a valid HRT prescription

For peer review only - http://bmjopen.bmj.com/site/about/guidelines.xhtml

Past user	A woman whose HRT prescription has expired and who does not renew the prescription within 30 days

HRT – hormone replacement therapy; CVD – cardiovascular disease

Meta-analysis

The HRs and Standard Errors (SEs) from these analyses will be pooled with identical analyses conducted on cohorts from Scotland and Wales. This two-stage analysis procedure will use random effects models to pool results across cohorts.

Sensitivity analyses

Confounding will primarily be addressed by entering a wide range of covariates into the Cox regression models. A number of additional sensitivity analyses will also be conducted to check for potential bias and sub-group effects including the following:

1. Analyses will be stratified into age categories of: 18 to <40, 40 to <55 and ≥55 years old. Women younger than 40 are more likely to have experience premature ovarian insufficiency whereas those 55 or over are likely to have experienced natural menopause.
2. An analysis of HRT after cancer diagnosis will be conducted using a new-user design i.e. restricting analyses to cancer patients who did not use HRT before diagnosis.
3. A negative control analysis will be conducted to check for unmeasured confounding by repeating the main analysis with death from other causes as the outcome because HRT is not expected to influence death from other causes (excluding cancer and cardiovascular disease).
4. It is possible that poor compliance may confound the analyses. To investigate this an analysis excluding individuals with only one prescription will be conducted, as patients continuing to be prescribed HRT are more likely to be adhering to their prescriptions.
5. A propensity score matched analysis will be conducted. First, logistic regression will be used to determine the propensity to be prescribed HRT. Matching will be implemented in Stata (Statacorp LLC, Texas, USA) to identify a HRT user group and a propensity score matched non-user group. Cox regression will then be used to determine the time to cancer-specific death in the two groups.

For peer review only - http://bmjopen.bmj.com/site/about/guidelines.xhtml

Missing data

Multiple imputation will be used to impute missing values for variables including BMI, smoking and alcohol status, stage and grade according to published guidelines[53]. As recommended, cancer-specific death status and cumulative hazard will be included in imputation models as will all variables included in regression models. The number of imputed datasets will correspond to the proportion of missing data and results will be combined using Rubin's rules[54].

Limitations

The completeness of stage data will be of importance to the analysis of HRT after diagnosis. Stage completeness is expected to be high for the majority of cancers we propose investigating including lung cancer (~90%); colorectal cancer (~90%) and malignant melanoma (~80%)[55]. For other sites and earlier years of diagnosis, however, stage is more likely to be missing, so it will be imputed by multiple imputation where necessary. Stage completeness is not expected to unduly influence the analysis of HRT before cancer diagnosis on survival and is arguably not appropriate, as it could lie on the causal pathway in these cases.

Smoking and BMI are well recorded in GP records but are not complete. Completeness for smoking is estimated to be approximately 95% in recent UK GP records and for BMI it is roughly 80%. Multiple imputation will be used to impute missing values for these factors.

There are several potential sources of bias that arise from a likely difference in prescribing patterns of HRT for women with cancer. An allocation bias may arise because women with more severe cancer are less likely to be prescribed HRT and therefore more likely to be allocated to the unexposed group. Alternatively, there may be a general aversion to prescribing HRT in this population, so that only those women whose cancers have a high chance of remission are considered for HRT. We will seek to address this in several ways, first, these concerns would not affect prescription patterns prior to cancer diagnosis, second, a 'new-user' sensitivity analysis will be conducted which excludes individuals with a history of HRT use prior to their cancer diagnosis. These analyses will provide a basis for assessing the potential impact of differing prescribing patterns and allocation bias.

Sample size calculation

For peer review only - http://bmjopen.bmj.com/site/about/guidelines.xhtml

Detectable hazard ratios were calculated based on case numbers from the English study cohort (Table 4). Statistical power to detect a difference (the beta value) was set at 90% and significance (the alpha value) at 5%. We estimated that 7% of female cancer patients would use HRT after diagnosis, consistent with published reports of roughly 5% HRT usage in a Swedish population based colorectal cancer cohort after diagnosis[56], and 9% HRT usage in a Danish melanoma cohort after diagnosis[57]. These calculations show that we would be able to detect HRs of 1.5 or greater with HRT use after diagnosis for most cancer sites.

For peer review only

For peer review only - http://bmjopen.bmj.com/site/about/guidelines.xhtml

Table 4. Detectable hazard ratios for each type of cancer assuming 90% power to detect a difference and statistical significance at p<0.05

		Cancer specific mortality				All-cause mortality			
		HRT prior to diagnosis[1]		HRT post diagnosis analysis[2]		HRT prior to diagnosis[1]		HRT post diagnosis analysis[2]	
Cancer type	Total cases	Total deaths	Detectable HR	Deaths >6 months from diagnosis	Detectable HR	Total deaths	Detectable HR	Deaths >6 months from diagnosis	Detectable HR
**Gastro-intestinal**									
Oral	5, 855	901	1.53 (or 0.65)	649	1.65 (or 0.61)	2,350	1.30 (or 0.74)	1,762	1.35 (or 0.74)
Oesophageal	11, 462	4,077	1.22 (or 0.82)	2,237	1.37 (or 0.73)	8,591	1.15 (or 0.87)	4,415	1.21 (or 0.83)
Gastric	5, 579	2,735	1.28 (or 0.78)	1,241	1.44 (or 0.70)	4,134	1,22 (or 0.82)	2,002	1.32 (or 0.75)
Pancreatic	9, 940	7,110	1.16 (or 0.86)	2,263	1.31 (or 0.77)	8,320	1.15 (or 0.87)	2,683	1.28 (or 0.78)
Liver	3, 984	2,405	1.30 (or 0.77)	813	1.57 (or 0.64)	3,182	1,25 (or 0.80)	1,068	1.48 (or 0.68)
Colorectal	37, 915	10,265	1.13 (or 0.88)	6,260	1.17 (or 0.85)	17,989	1.10 (or 0.91)	11,418	1.13 (or 0.89)
**Other**									
Lung	40, 084	24,904	1.08 (or 0.92)	10,582	1.13 (or 0.88)	30,450	1.08 (or 0.93)	13,250	1.12 (or 0.90)
Melanoma	16, 382	1,059	1.48 (or 0.68)	902	1.53 (or 0.65)	2,558	1.29 (or 0.78)	2,277	1.31 (or 0.77)
Thyroid	3, 876	321	2.04 (or 0.49)	143	2.89 (or 0.35)	554	1.72 (or 0.58)	325	2.04 (or 0.49)

For peer review only - http://bmjopen.bmj.com/site/about/guidelines.xhtml

Brain	4, 759	2,422	1.29 (or 0.77)	1,059	1.48 (or 0.68)	3,319	1.25 (or 0.80)	1,362	1.41 (or 0.71)
**Haematological**									
Leukaemia	8, 620	2,593	1.28 (or 0.78)	1,258	1.43 (or 0.70)	4,326	1.21 (or 0.82)	2,494	1.29 (or 0.78)
Multiple myeloma	5, 381	1,753	1.36 (or 0.74)	1,251	1.43 (or 0.70)	2,842	1.27 (or 0.79)	2,022	1.33 (or 0.75)
Non-Hodgkin's lymphoma	12, 584	2,920	1.26 (or 0.79)	1,665	1.37 (or 0.73)	5,166	1.19 (or 0.84)	3,218	1.25 (or 0.80)
**Gynaecological**									
Cervical	5, 594	1,093	1.47 (or 0.68)	784	1.57 (or 0.63)	1,753	1.36 (or 0.74)	1,265	1.43 (or 0.70)
Endometrial	18, 855	2,913	1.27 (or 0.79)	2,188	1.31 (or 0.76)	5,821	1.18 (or 0.85)	4,557	1.21 (or 0.83)
Ovarian	16, 526	6,444	1.17 (or 0.85)	2,153	1.22 (or 0.82)	8,953	1.14 (or 0.87)	5,793	1.18 (or 0.85)
**Urinary**									
Renal	7,895	2,153	1.32 (or 0.76)	1,167	1.45 (or 0.69)	3,537	1.24 (or 0.81)	2,124	1.32 (or 0.76)
Bladder	10, 507	2,256	1.31 (or 0.76)	1,301	1.42 (or 0.70)	4,440	1.21 (or 0.83)	3,068	1.26 (or 0.79)

HRT – Hormone Replacement Therapy; HR – Hazard Ratio

1. Uses total number of deaths; 2. Uses number of deaths more than 6 months after diagnosis only

For peer review only

For peer review only - http://bmjopen.bmj.com/site/about/guidelines.xhtml

Ethics and dissemination

Ethical approval for this project was obtained from the QResearch scientific committee [Ref: OX24, project title “Use of hormone replacement therapy and survival from cancer”]. Ethical approval for the QResearch database is obtained annually from East Midlands - Derby Research Ethics Committee [Ref: 18/EM/0400].

The findings of this study will be submitted for peer-reviewed publication in an academic journal. In addition, the findings will be provided to Cancer Research UK for public dissemination, with the assistance of the Patient and Public Involvement (PPI) panel.

Patient and public involvement

The project will be supported throughout by a PPI panel, made up of women with experience of non-oestrogenic cancers. Members of the Northern Ireland Cancer Research Consumer Forum (NICRCF), a cancer patients’ group, provided feedback on the project proposal before submission to CRUK.

Another PPI panel of four women with a previous diagnosis of cancer was convened to inform the English component of this project (i.e. the one presented in this protocol). They agreed the study was important and potentially valuable for female cancer survivors who experience menopausal symptoms. The panel suggested that both cancer-specific mortality and subsequent cancer diagnoses would be of interest to women in this demographic considering whether to use HRT. Future discussions will seek feedback on the relevance and resonance of findings and guide us on research communications and dissemination.

For peer review only

References

1. Hamoda H, Panay N, Arya R, et al. The British Menopause Society & Women's Health Concern 2016 recommendations on hormone replacement therapy in menopausal women. *Post Reproductive Health* 2016;22(4):165-83. doi: 10.1177/2053369116680501
2. Goodman NF, Cobin RH, Ginzburg SB, et al. American Association of Clinical Endocrinologists Medical Guidelines for Clinical Practice for the diagnosis and treatment of menopause: executive summary of recommendations. *Endocr Pract* 2011;17(6):949-54. doi: 10.4158/ep.17.6.949 [published Online First: 2011/12/24]
3. Menon U, Burnell M, Sharma A, et al. Decline in use of hormone therapy among postmenopausal women in the United Kingdom. *Menopause* 2007;14(3 Pt 1):462-7. doi: 10.1097/01.gme.0000243569.70946.9d [published Online First: 2007/01/24]
4. Ameye L, Antoine C, Paesmans M, et al. Menopausal hormone therapy use in 17 European countries during the last decade. *Maturitas* 2014;79(3):287-91. doi: 10.1016/j.maturitas.2014.07.002
5. Million Women Study collaborators. Breast cancer and hormone-replacement therapy in the Million Women Study. *The Lancet* 2003;362(9382):419-27. doi: https://doi.org/10.1016/S0140-6736(03)14065-2
6. Cagnacci A, Venier M. The Controversial History of Hormone Replacement Therapy. *Medicina (Kaunas)* 2019;55(9) doi: 10.3390/medicina55090602 [published Online First: 2019/09/22]
7. Writing Group for the Women's Health Initiative I. Risks and Benefits of Estrogen Plus Progestin in Healthy Postmenopausal WomenPrincipal Results From the Women's Health Initiative Randomized Controlled Trial. *JAMA* 2002;288(3):321-33. doi: 10.1001/jama.288.3.321
8. Marjoribanks J, Farquhar C, Roberts H, et al. Long-term hormone therapy for perimenopausal and postmenopausal women. *Cochrane Database Syst Rev* 2017;1:CD004143. doi: 10.1002/14651858.CD004143.pub5 [published Online First: 2017/01/18]
9. Shen SS, Smith CL, Hsieh JT, et al. Expression of estrogen receptors-α and -β in bladder cancer cell lines and human bladder tumor tissue. *Cancer* 2006;106(12):2610-16. doi: 10.1002/cncr.21945
10. Wang X, Chen Q, Huang X, et al. Effects of 17β-estradiol and tamoxifen on gastric cancer cell proliferation and apoptosis and ER-α36 expression. *Oncol Lett* 2017;13(1):57-62. doi: 10.3892/ol.2016.5424 [published Online First: 2017/01/27]
11. Sukocheva OA, Wee C, Ansar A, et al. Effect of estrogen on growth and apoptosis in esophageal adenocarcinoma cells. *Dis Esophagus* 2013;26(6):628-35. doi: 10.1111/dote.12000 [published Online First: 2012/11/21]
12. Kanda N, Watanabe S. 17β-estradiol, Progesterone, and Dihydrotestosterone Suppress the Growth of Human Melanoma by Inhibiting Interleukin-8 Production. *Journal of Investigative Dermatology* 2001;117(2):274-83. doi: https://doi.org/10.1046/j.1523-1747.2001.01422.x
13. Hammoud Z, Tan B, Badve S, et al. Estrogen promotes tumor progression in a genetically defined mouse model of lung adenocarcinoma. *Endocr Relat Cancer* 2008;15(2):475-83. doi: 10.1677/erc-08-0002 [published Online First: 2008/05/30]
14. Ge H, Yan Y, Tian F, et al. Prognostic value of estrogen receptor α and estrogen receptor β in gastric cancer based on a meta-analysis and The Cancer Genome Atlas (TCGA) datasets. *Int J Surg* 2018;53:24-31. doi: 10.1016/j.ijsu.2018.03.027 [published Online First: 2018/03/21]
15. Cook MB, McGlynn KA, Devesa SS, et al. Sex disparities in cancer mortality and survival. *Cancer Epidemiol Biomarkers Prev* 2011;20(8):1629-37. doi: 10.1158/1055-9965.Epi-11-0246 [published Online First: 2011/07/14]
16. Radkiewicz C, Johansson ALV, Dickman PW, et al. Sex differences in cancer risk and survival: A Swedish cohort study. *Eur J Cancer* 2017;84:130-40. doi: 10.1016/j.ejca.2017.07.013 [published Online First: 2017/08/15]

For peer review only - http://bmjopen.bmj.com/site/about/guidelines.xhtml

17. Kim HW, Kim JH, Lim BJ, et al. Sex Disparity in Gastric Cancer: Female Sex is a Poor Prognostic Factor for Advanced Gastric Cancer. *Ann Surg Oncol* 2016;23(13):4344-51. doi: 10.1245/s10434-016-5448-0 [published Online First: 2016/11/03]
18. McGlynn KA, Hagberg K, Chen J, et al. Menopausal hormone therapy use and risk of primary liver cancer in the clinical practice research datalink. *International journal of cancer* 2016;138(9):2146-53. doi: 10.1002/ijc.29960 [published Online First: 2015/12/31]
19. Hassan MM, Botrus G, Abdel-Wahab R, et al. Estrogen Replacement Reduces Risk and Increases Survival Times of Women With Hepatocellular Carcinoma. *Clin Gastroenterol Hepatol* 2017;15(11):1791-99. doi: 10.1016/j.cgh.2017.05.036 [published Online First: 2017/06/01]
20. Simin J, Tamimi R, Lagergren J, et al. Menopausal hormone therapy and cancer risk: An overestimated risk? *European Journal of Cancer* 2017;84:60-68. doi: 10.1016/j.ejca.2017.07.012
21. Grodstein F, Newcomb PA, Stampfer MJ. Postmenopausal hormone therapy and the risk of colorectal cancer: a review and meta-analysis. *Am J Med* 1999;106(5):574-82. doi: 10.1016/s0002-9343(99)00063-7 [published Online First: 1999/05/21]
22. Mørch LS, Lidegaard Ø, Keiding N, et al. The influence of hormone therapies on colon and rectal cancer. *Eur J Epidemiol* 2016;31(5):481-9. doi: 10.1007/s10654-016-0116-z [published Online First: 2016/01/14]
23. Lope V, Fernández de Larrea N, Pérez-Gómez B, et al. Menstrual and Reproductive Factors and Risk of Gastric and Colorectal Cancer in Spain. *PLoS One* 2016;11(10):e0164620. doi: 10.1371/journal.pone.0164620 [published Online First: 2016/10/25]
24. Benson VS, Kirichek O, Beral V, et al. Menopausal Hormone Therapy and Risk of Central Nervous System Tumours: a UK-based Nested Case-Control Study. *International Journal of Epidemiology* 2015;44(suppl_1):i52-i52. doi: 10.1093/ije/dyv097.198
25. Jin C, Lang B. Hormone replacement therapy and lung cancer risk in women: a meta-analysis of cohort studies: Hormone replacement therapy and lung cancer risk. *Medicine (Baltimore)* 2019;98(51):e17532-e32. doi: 10.1097/MD.0000000000017532
26. Bae J-M, Kim EH. Hormonal Replacement Therapy and the Risk of Lung Cancer in Women: An Adaptive Meta-analysis of Cohort Studies. *J Prev Med Public Health* 2015;48(6):280-86. doi: 10.3961/jpmph.15.054 [published Online First: 2015/11/09]
27. Kane EV, Bernstein L, Bracci PM, et al. Postmenopausal hormone therapy and non-Hodgkin lymphoma: a pooled analysis of InterLymph case-control studies. *Ann Oncol* 2013;24(2):433-41. doi: 10.1093/annonc/mds340 [published Online First: 2012/09/13]
28. Costas L, Lujan-Barroso L, Benavente Y, et al. Reproductive Factors, Exogenous Hormone Use, and Risk of B-Cell Non-Hodgkin Lymphoma in a Cohort of Women From the European Prospective Investigation Into Cancer and Nutrition. *Am J Epidemiol* 2019;188(2):274-81. doi: 10.1093/aje/kwy259 [published Online First: 2018/11/28]
29. Morton LM, Wang SS, Richesson DA, et al. Reproductive factors, exogenous hormone use and risk of lymphoid neoplasms among women in the National Institutes of Health-AARP Diet and Health Study Cohort. *Int J Cancer* 2009;124(11):2737-43. doi: 10.1002/ijc.24248 [published Online First: 2009/03/03]
30. Daugherty SE, Lacey JJV, Pfeiffer RM, et al. Reproductive factors and menopausal hormone therapy and bladder cancer risk in the NIH-AARP Diet and Health Study. *International Journal of Cancer* 2013;133(2):462-72. doi: 10.1002/ijc.28022
31. Deli T, Orosz M, Jakab A. Hormone Replacement Therapy in Cancer Survivors – Review of the Literature. *Pathology & Oncology Research* 2020;26(1):63-78. doi: 10.1007/s12253-018-00569-x
32. Webber L, Anderson RA, Davies M, et al. HRT for women with premature ovarian insufficiency: a comprehensive review. *Hum Reprod Open* 2017;2017(2):hox007. doi: 10.1093/hropen/hox007 [published Online First: 2017/07/12]

For peer review only - http://bmjopen.bmj.com/site/about/guidelines.xhtml

33. Edey KA, Rundle S, Hickey M. Hormone replacement therapy for women previously treated for endometrial cancer. *Cochrane Database of Systematic Reviews* 2018(5) doi: 10.1002/14651858.CD008830.pub3
34. Ji J, Sundquist J, Sundquist K. Use of hormone replacement therapy improves the prognosis in patients with colorectal cancer: A population-based study in Sweden. *Int J Cancer* 2018;142(10):2003-10. doi: 10.1002/ijc.31228 [published Online First: 2017/12/23]
35. Chan JA, Meyerhardt JA, Chan AT, et al. Hormone replacement therapy and survival after colorectal cancer diagnosis. *J Clin Oncol* 2006;24(36):5680-6. doi: 10.1200/jco.2006.08.0580 [published Online First: 2006/12/21]
36. Jang YC, Huang HL, Leung CY. Association of hormone replacement therapy with mortality in colorectal cancer survivor: a systematic review and meta-analysis. *BMC Cancer* 2019;19(1):1199. doi: 10.1186/s12885-019-6428-0 [published Online First: 2019/12/11]
37. MacKie RM, Bray CA. Hormone replacement therapy after surgery for stage 1 or 2 cutaneous melanoma. *Br J Cancer* 2004;90(4):770-2. doi: 10.1038/sj.bjc.6601595 [published Online First: 2004/02/19]
38. Krul IM, Opstal-van Winden AWJ, Aleman BMP, et al. Breast Cancer Risk After Radiation Therapy for Hodgkin Lymphoma: Influence of Gonadal Hormone Exposure. *Int J Radiat Oncol Biol Phys* 2017;99(4):843-53. doi: 10.1016/j.ijrobp.2017.07.016 [published Online First: 2017/09/11]
39. Yang X, Wang C, He X, et al. Hormone therapy for premature ovarian insufficiency patients with malignant hematologic diseases. *Climacteric* 2017;20(3):268-73. doi: 10.1080/13697137.2017.1309382 [published Online First: 2017/04/13]
40. Ganti AK. Another nail in the coffin for hormone-replacement therapy? *The Lancet* 2009;374(9697):1217-18. doi: https://doi.org/10.1016/S0140-6736(09)61571-3
41. Biglia N, Gadducci A, Ponzone R, et al. Hormone replacement therapy in cancer survivors. *Maturitas* 2004;48(4):333-46. doi: https://doi.org/10.1016/j.maturitas.2003.09.031
42. Cobin RH, Goodman NF, Committee ARES. American Association of Clinical Endocrinologists and American College of Endocrinology position statement on menopause–2017 update. *Endocrine practice* 2017;23(7):869-80.
43. National Institute for Health and Care Excellence. Menopause: diagnosis and management2019.
44. Welton AJ, Vickers MR, Kim J, et al. Health related quality of life after combined hormone replacement therapy: randomised controlled trial. *BMJ* 2008;337:a1190. doi: 10.1136/bmj.a1190
45. Oliver-Williams C, Glisic M, Shahzad S, et al. The route of administration, timing, duration and dose of postmenopausal hormone therapy and cardiovascular outcomes in women: a systematic review. *Hum Reprod Update* 2019;25(2):257-71. doi: 10.1093/humupd/dmy039 [published Online First: 2018/12/07]
46. Kontopantelis E, Stevens RJ, Helms PJ, et al. Spatial distribution of clinical computer systems in primary care in England in 2016 and implications for primary care electronic medical record databases: a cross-sectional population study. *BMJ Open* 2018;8(2):e020738. doi: 10.1136/bmjopen-2017-020738
47. Hippisley-Cox J, Stables D Fau - Pringle M, Pringle M. QRESEARCH: a new general practice database for research. (1476-0320 (Print))
48. British Medical Association Joint Formulary Committee Pharmaceutical Society (Great Britain). British national formulary: Pharmaceutical Pr 2003.
49. Vinogradova Y, Coupland C, Hippisley-Cox J. Use of hormone replacement therapy and risk of venous thromboembolism: nested case-control studies using the QResearch and CPRD databases. *Bmj* 2019 doi: 10.1136/bmj.k4810

For peer review only - http://bmjopen.bmj.com/site/about/guidelines.xhtml

50. Arık A, Dodd E, Streftaris G. Cancer morbidity trends and regional differences in England—A Bayesian analysis. *PLOS ONE* 2020;15(5):e0232844. doi: 10.1371/journal.pone.0232844
51. Public Health England. Linking treatment tables - chemotherapy, tumour resections and radiotherapy. 4.4 ed: Cancer Research UK - Public Health England, 2018:58.
52. Suissa S, Azoulay L. Metformin and the risk of cancer: time-related biases in observational studies. *Diabetes Care* 2012;35(12):2665-73. doi: 10.2337/dc12-0788 [published Online First: 2012/11/23]
53. White IR, Royston P, Wood AM. Multiple imputation using chained equations: Issues and guidance for practice. *Stat Med* 2011;30(4):377-99. doi: 10.1002/sim.4067 [published Online First: 2011/01/13]
54. Rubin DB. Multiple imputation for nonresponse in surveys: John Wiley & Sons 2004.
55. Registries UKaIAoC. UKIACR: Performance Indicators 2018 Report, 2018.
56. Ji J, Sundquist J, Sundquist K. Use of hormone replacement therapy improves the prognosis in patients with colorectal cancer: A population-based study in Sweden. *International Journal of Cancer* 2018;142(10):2003-10. doi: 10.1002/ijc.31228
57. Hicks B, Kristensen KB, Pedersen SA, et al. Hormone replacement therapy and the risk of melanoma in post-menopausal women. *Human Reproduction* 2019;34(12):2418-29.

For peer review only

For peer review only - http://bmjopen.bmj.com/site/about/guidelines.xhtml

Author's contributions

All authors were involved in the preparation of this manuscript (TR, JB, AC, WM, CCo, PST, SD, CCa, JHC).

CCa conceived the study, and TR, JB, AC, WM, PST, CCa & JHC made substantial contributions to the study design. TR, CCo, CCa and JHC made substantial contributions to the acquisition and analysis of data.

TR drafted the original manuscript. JB, AC, WM, CCo, PST, SD, CCa & JHC provided critical revision of the manuscript for major intellectual content.

All authors have approved the final version of this manuscript for publication and agree to be accountable for all aspects of the work in ensuring that questions related to the accuracy or integrity of any part of the work are appropriately investigated and resolved.

Sources of funding

This work was supported by Cancer Research UK [grant number: C37316/A29656]

The study sponsor has no role in: study design; collection, management, analysis or interpretation of data; writing of the report; or the decision to submit the report for publication.

JHC has received grants from the National Institute for Health Research Biomedical Research Centre, Oxford, John Fell Oxford University Press Research Fund, Cancer Research UK (grant number C5255/A18085) through the Cancer Research UK Oxford Centre, and the Oxford Wellcome Institutional Strategic Support Fund (204826/Z/16/Z) during the conduct of the study.

AC is supported by a Clinical Research Training Fellowship from Cancer Research UK (DCS-CRUK-CRTF20-AC).

Competing interests statement

JHC is an unpaid director of the Qresearch database (partnership between the University of Oxford and EMIS, commercial supplier of GP computer systems). She is shareholder and former director of ClinRisk Ltd outside the submitted work.

PST reports consulting with AstraZeneca and Duke-NUS outside the submitted work.

The remaining authors declare no competing interests.

Word count

6,748

For peer review only - http://bmjopen.bmj.com/site/about/guidelines.xhtml

STROBE 2007 (v4) Statement—Checklist of items that should be included in reports of *cohort studies*

Section/Topic	Item #	Recommendation	Reported on page #
**Title and abstract**	1	(*a*) Indicate the study's design with a commonly used term in the title or the abstract	1
		(*b*) Provide in the abstract an informative and balanced summary of what was done and what was found	2
**Introduction**			
Background/rationale	2	Explain the scientific background and rationale for the investigation being reported	4-6
Objectives	3	State specific objectives, including any prespecified hypotheses	7
**Methods**			
Study design	4	Present key elements of study design early in the paper	7
Setting	5	Describe the setting, locations, and relevant dates, including periods of recruitment, exposure, follow-up, and data collection	7
Participants	6	(*a*) Give the eligibility criteria, and the sources and methods of selection of participants. Describe methods of follow-up	7
		(*b*) For matched studies, give matching criteria and number of exposed and unexposed	n/a
Variables	7	Clearly define all outcomes, exposures, predictors, potential confounders, and effect modifiers. Give diagnostic criteria, if applicable	8-13
Data sources/ measurement	8*	For each variable of interest, give sources of data and details of methods of assessment (measurement). Describe comparability of assessment methods if there is more than one group	8-13
Bias	9	Describe any efforts to address potential sources of bias	16, 17
Study size	10	Explain how the study size was arrived at	18, 19
Quantitative variables	11	Explain how quantitative variables were handled in the analyses. If applicable, describe which groupings were chosen and why	13- 15
Statistical methods	12	(*a*) Describe all statistical methods, including those used to control for confounding	13- 15
		(*b*) Describe any methods used to examine subgroups and interactions	14- 16
		(*c*) Explain how missing data were addressed	17
		(*d*) If applicable, explain how loss to follow-up was addressed	n/a
		(*e*) Describe any sensitivity analyses	16

For peer review only

**Results – CHECKLIST NOT COMPLETED BEYOND THIS POINT AS THE CURRENT SUBMISSION IS A PROTOCOL ONLY**			
Participants	13*	(a) Report numbers of individuals at each stage of study—eg numbers potentially eligible, examined for eligibility, confirmed eligible, included in the study, completing follow-up, and analysed	n/a
		(b) Give reasons for non-participation at each stage	n/a
		(c) Consider use of a flow diagram	n/a
Descriptive data	14*	(a) Give characteristics of study participants (eg demographic, clinical, social) and information on exposures and potential confounders	n/a
		(b) Indicate number of participants with missing data for each variable of interest	n/a
		(c) Summarise follow-up time (eg, average and total amount)	n/a
Outcome data	15*	Report numbers of outcome events or summary measures over time	n/a
Main results	16	(*a*) Give unadjusted estimates and, if applicable, confounder-adjusted estimates and their precision (eg, 95% confidence interval). Make clear which confounders were adjusted for and why they were included	n/a
		(*b*) Report category boundaries when continuous variables were categorized	n/a
		(*c*) If relevant, consider translating estimates of relative risk into absolute risk for a meaningful time period	n/a
Other analyses	17	Report other analyses done—eg analyses of subgroups and interactions, and sensitivity analyses	n/a
**Discussion**			
Key results	18	Summarise key results with reference to study objectives	n/a
**Limitations**			
Interpretation	20	Give a cautious overall interpretation of results considering objectives, limitations, multiplicity of analyses, results from similar studies, and other relevant evidence	n/a
Generalisability	21	Discuss the generalisability (external validity) of the study results	n/a
**Other information**			
Funding	22	Give the source of funding and the role of the funders for the present study and, if applicable, for the original study on which the present article is based	n/a

*Give information separately for cases and controls in case-control studies and, if applicable, for exposed and unexposed groups in cohort and cross-sectional studies.

Note: An Explanation and Elaboration article discusses each checklist item and gives methodological background and published examples of transparent reporting. The STROBE checklist is best used in conjunction with this article (freely available on the Web sites of PLoS Medicine at http://www.plosmedicine.org/, Annals of Internal Medicine at http://www.annals.org/, and Epidemiology at http://www.epidem.com/). Information on the STROBE Initiative is available at www.strobe-statement.org.

For peer review only
