# Supplementary material for: Hormone replacement therapy and cancer survival: a longitudinal cohort study: protocol paper
Source: BMJ Open. 2021 Aug 2;11(8):e046701. doi: 10.1136/bmjopen-2020-046701 (PMC8330592; doi:10.1136/bmjopen-2020-046701)
Supplement: Reviewer comments [file bmjopen-2020-046701.reviewer_comments.pdf]

PEER REVIEW HISTORY

BMJ Open publishes all reviews undertaken for accepted manuscripts. Reviewers are asked to complete a checklist review form (**http://bmjopen.bmj.com/site/about/resources/checklist.pdf**) and are provided with free text boxes to elaborate on their assessment. These free text comments are reproduced below.

ARTICLE DETAILS

**TITLE (PROVISIONAL)**	Hormone Replacement Therapy and Cancer Survival: A Longitudinal Cohort Study - protocol paper
**AUTHORS**	Ranger, Tom; Burchardt, Judith; Clift, Ashley; Mei, Xue; Coupland, Carol; Tan, Pui San; Dixon, Sharon; Cardwell, Christopher; Hippisley-Cox, Julia

This article was not externally reviewed at BMJ Open. Protocols that have been independently assessed prior to submission to BMJ Open are usually fast-tracked to publication on the grounds that further substantial changes will not be possible. This independent assessment will usually be external, independent review for both a substantial grant award from a non-commercial or government funder and ethics approval.
